# Changing behaviour, ‘more or less’: do implementation and de-implementation interventions include different behaviour change techniques?

**DOI:** 10.1186/s13012-021-01089-0

**Published:** 2021-02-25

**Authors:** Andrea M. Patey, Jeremy M. Grimshaw, Jill J. Francis

**Affiliations:** 1 School of Health Sciences, City, University of London, 10 Northampton Square, London, EC1V 0HB UK; 2 Centre of Implementation Research, Ottawa Hospital Research Institute – General Campus, 501 Smyth Road, Ottawa, Ontario K1H 8L6 Canada; 3 Faculty of Medicine, University of Ottawa, 451 Smyth Road, Ottawa, Ontario K1H 8M5 Canada; 4 School of Health Sciences, University of Melbourne, Melbourne, Victoria 3010 Australia

**Keywords:** De-implementation versus implementation, Behaviour change, Intervention content, Techniques, Taxonomy, Intervention design

## Abstract

**Background:**

Decreasing ineffective or harmful healthcare practices (de-implementation) may require different approaches than those used to promote uptake of effective practices (implementation). Few psychological theories differentiate between processes involved in decreasing, versus increasing, behaviour. However, it is unknown whether implementation and de-implementation interventions already use different approaches. We used the behaviour change technique (BCT) taxonomy (version 1) (which includes 93 BCTs organised into 12 groupings) to investigate whether implementation and de-implementation interventions for clinician behaviour change use different BCTs.

**Methods:**

Intervention descriptions in 181 articles from three systematic reviews in the Cochrane Library were coded for (a) implementation versus de-implementation and (b) intervention content (BCTs) using the BCT taxonomy (v1). BCT frequencies were calculated and compared using Pearson’s chi-squared (*χ*^2^), Yates’ continuity correction and Fisher’s exact test, where appropriate. Identified BCTs were ranked according to frequency and rankings for de-implementation versus implementation interventions were compared and described.

**Results:**

Twenty-nine and 25 BCTs were identified in implementation and de-implementation interventions respectively. *Feedback on behaviour* was identified more frequently in implementation than de-implementation (*Χ*^*2*^(2, *n*=178) = 15.693, *p* = .000057). Three BCTs were identified more frequently in de-implementation than implementation: *Behaviour substitution* (*Χ*^*2*^(2, *n*=178) = 14.561, *p* = .0001; Yates’ continuity correction); *Monitoring of behaviour by others without feedback* (*Χ*^*2*^(2, *n*=178) = 16.187, *p* = .000057; Yates’ continuity correction); and *Restructuring social environment* (*p* = .000273; Fisher’s 2-sided exact test).

**Conclusions:**

There were some significant differences between BCTs reported in implementation and de-implementation interventions suggesting that researchers may have implicit theories about different BCTs required for de-implementation and implementation. These findings do not imply that the BCTs identified as targeting implementation or de-implementation are effective, rather simply that they were more frequently used. These findings require replication for a wider range of clinical behaviours. The continued accumulation of additional knowledge and evidence into whether implementation and de-implementation is different will serve to better inform researchers and, subsequently, improve methods for intervention design.

**Supplementary Information:**

The online version contains supplementary material available at 10.1186/s13012-021-01089-0.

Contribution to literature

- De-implementation to decrease ineffective or harmful healthcare practices may require different approaches than implementation used to promote uptake of new procedures. However, there is little to no guidance on how to de-implement low-value or harmful healthcare practices or what types of interventions are better suited for de-implementation.
- Investigation of the behaviour change techniques (BCTs) used in implementation and de-implementation interventions to identify whether intervention components are different will clarify the content of current interventions and may help to explain a theoretical base for designing de-implementation interventions.
- There were some significant differences between BCTs reported in implementation and de-implementation interventions suggesting that researchers may have implicit theories about different BCTs required for de-implementation and implementation. These findings do not imply that the BCTs identified as targeting implementation or de-implementation are effective, rather simply that they were more frequently used.
- The continued accumulation of knowledge and evidence into whether implementation and de-implementation differ will serve to better inform researchers and improve methods for intervention design.

## Introduction

The problem of overuse of ineffective or harmful care which can lead to poor patient outcomes due to adverse events of treatments or unwarranted secondary tests and inefficient use of scarce healthcare resources threatening the sustainability of healthcare systems, has led to an increasing need to identify effective de-implementation interventions of low-value care [1–3]. For example, the BMJ’s Too Much Medicine campaign noted that identifying strategies to reduce unnecessary tests, diagnoses and treatments (i.e. de-implementation) will benefit patients by directly avoiding harm and helping create a more sustainable healthcare system [4, 5]. Projects such as Choosing Wisely have noted the importance of addressing de-implementation strategies to improve the care patients receive whilst eliminating wasteful spending [6–9]. Policy interest in de-implementation with programmes such as Choosing Wisely and Too Much Medicine campaigns advocate for more effective de-implementation interventions but remain unclear what the effective strategies should be.

Decreasing ineffective or harmful healthcare practices (de-implementation) may require different approaches than those used to promote uptake of new procedures (implementation) [10, 11]. However, there is little to no guidance on how to de-implement low-value or harmful healthcare practices or what types of interventions are better suited for de-implementation [2]. Investigation into the theoretical basis for designing implementation and de-implementation interventions differently has indicated that theories of behaviour change generally do not distinguish between implementation and de-implementation [12]. The one theory that does make the distinction (Operant Learning Theory [13]) may be difficult to apply in healthcare settings [12].

The term de-implementation is relatively new in the field of implementation research, having been discussed for only the last 8 years [10, 11, 14–16]. However, researchers have been designing implementation and de-implementation interventions for decades but rarely explicitly distinguished between them. It is unclear what approaches are being used and whether implementation and de-implementation interventions do require different strategies. It is thus important to describe the content of both implementation and de-implementation interventions and unpack their ‘active ingredients’ or behaviour change techniques to (1) determine what is actually included in both kinds of interventions and (2) provide a theoretical perspective to inform guidance for designing de-implementation interventions.

Behaviour change techniques (BCTs) are defined as ‘observable, replicable and irreducible components of an intervention designed to alter or redirect causal processes that regulate behaviour; that is, a technique is proposed to be an “active ingredient”’ [17]. Taxonomies of BCTs provide standard definitions and labels for intervention components that allow for systematic comparison of common components in intervention descriptions from a variety of clinical settings, behaviours and professional groups. The most comprehensive taxonomy is the BCT taxonomy (version 1), which consists of 93 techniques [17] which are hierarchically organised into 16 groupings. Each technique has a definition and an example to aid in designing interventions or coding of pre-existing intervention descriptions. The majority of the examples provided within the taxonomy are directed at changing health behaviours of patients and members of the public but can and have been applied to describe behaviour change interventions relating to clinical practice. Whilst there are a number of taxonomies that permit the identification of intervention components such as the Expert Recommendations of Implementation Strategies (ERIC) [18] or the Effective Practice and Organisation of Care (EPOC) [19] taxonomies, these taxonomies do not possess the granularity and specificity the BCT taxonomy contains. This granularity permits for an in-depth investigation of the potentially subtle differences in implementation and de-implementation that may be overlooked with other taxonomies.

Investigation of the BCTs used in implementation and de-implementation interventions to identify whether they are different will clarify the content of current interventions and may help to explain a theoretical base for designing de-implementation interventions. The objectives of the current study were to use the BCT taxonomy (v1) [17] to (1) determine what intervention components are included in both kinds of interventions and (2) whether these components differ.

## Methods

### Design and review selection

We conducted a secondary analysis of a subset of intervention studies included in three Cochrane systematic reviews. We sampled from systematic reviews completed under the auspices of the Cochrane Effective Practice and Organisation of Care group in the Cochrane Library. Titles, abstracts and plain language summaries of EPOC systematic reviews were screened for inclusion. The key eligibility criterion was that the reviews described the behaviour change as a change in frequency. We identified 142 potentially eligible EPOC systematic reviews and protocols on the date of extraction. Of these, 48 were protocols and 4 were withdrawn. Three reviews contained no studies. Of the 87 remaining reviews, 31 were excluded because the abstract or summary did not clearly indicate that health professionals were participants or report health professional behaviour as a study outcome. For example, in the review, ‘Effectiveness of intermediate care in nursing-led inpatients units’ the intervention focused on having nurses, rather than physicians, manage inpatient units; the behaviour was not a change in frequency (change in HCP role). Similarly, in the review titled ‘Dietary advice given by a dietician versus other health professionals or self-help to reduce blood cholesterol’, the outcomes were reported as patient blood cholesterol, body weight and high-density lipoprotein cholesterol levels, which were not measures of health professional behaviours.

Pragmatically, it would not have been possible to include studies from 56 reviews. Through a purposive selection process, reviews selected for inclusion in this study were *Audit and feedback: effects on professional practice and healthcare outcomes* [20], *Interventions for improving antibiotic prescribing practices for hospital inpatients* [21] and *Interventions for improving the appropriate use of imaging in people with musculoskeletal conditions* [22]. Criteria for purposive selection were (1) the reviews selected should include interventions that may target both implementation and de-implementation (i.e. increasing the frequency of high-value clinical behaviours and decreasing the frequency of low-value clinical behaviours) and (2) the reviews selected should not be limited to one professional group or setting but include various clinical settings and healthcare professions (e.g. primary care physicians, nurses, internists and other healthcare professionals in secondary and tertiary care facilities) to diversify the population of healthcare professional groups.

### Screening and selection of articles

Titles and abstracts of the included studies from the systematic reviews were screened for eligibility. Studies were excluded if the intervention was not delivered directly to healthcare professionals, the behaviour change was not described as a change in frequency, or the desired change in frequency was unclear. In instances where eligibility was unclear, the articles were retained for full-text screening. The same eligibility criteria were applied in screening of full-text articles.

### Inter-rater reliability

Two members of the research team (CSH, JJF) independently applied the inclusion criteria of 10% randomly selected articles initially screened by lead researcher (AMP). Agreement (Cohen’s kappa; *κ*) [23, 24] was calculated and any discrepancies were discussed with the lead researcher to clarify the coding of behaviour change frequency and to better inform the screening of remaining articles.

### Data extraction

Data extracted from the articles included (1) desired change in behaviour frequency (implementation versus de-implementation); (2) health professional group (research participants); (3) sample size; (4) study design; (5) description of behaviour; (6) presence or absence of control group (*y*/*n*); and (7) intervention details as published.

### Coding of intervention descriptions into behaviour change techniques

Using the BCT taxonomy (v1) definitions and examples from the coding manual [17], one researcher (AMP, who had completed formal training in BCT coding) coded the published intervention descriptions in all the included studies. Coding was conducted by assigning a BCT label from the taxonomy to passages of text of the intervention description from the article. All 93 techniques were considered for each intervention description. Content was coded for both the active intervention and the control condition. Two independent researchers with expertise in health psychology and training in the BCT taxonomy (v1) (HM and KM) coded the intervention descriptions of a subset of the included articles (20% each). Five coding assumptions were made to ensure consistency throughout the coding (see Table 1). Inter-rater reliability for identifying the same BCTs from intervention descriptions was assessed using percentage agreement; agreement above 80% was considered ‘satisfactory’ [25, 26]. Discrepancies between raters were resolved through discussion or consultation with a member of the BCT taxonomy research team (JJF).

**Table 1:** Coding assumptions for identifying behaviour change techniques [17] in intervention descriptions

Coding assumption	Behaviour change technique	BCT definition (contained in the Supplemental Materials)
Changes to hospital policies were coded as *Instruction on how to perform the behaviour* whether it was clear or not that HCPs were instructed on the new policy	*Instruction on how to perform the behaviour*	Advise or agree on how to perform the behaviour
If distribution of guidelines related to the behaviour were part of, or occurred during, the intervention both *Instruction on how to perform the behaviour* (see above) and *Information about health consequences* were coded.	*Information about health consequences*	Provide information (e.g. written, verbal, visual) about health consequences of performing the behaviour
Removal of drugs from a pharmacy list was coded as *Restructuring physical environment* despite the physical location of the drug removal was away from the HCP performing the behaviour. There was no BCT to reflect the removal of object similar to the BCT for *Adding object to the environment.*	*Restructuring physical environment*	Change or advise to change the physical environment in order to facilitate the performance of a wanted behaviour or create barriers to the unwanted behaviours (other than prompts/cues, rewards and punishments)
Interventions in which HCPs were required to discuss care with other colleagues or obtain signing authority for a test or treatment were coded as both *Social support* (*practical*) and *Restructuring social environment*.	*Social support* (*practical*) and *Restructuring social Envirunment *	Advise or arrange or provide practical help (e.g. from friends, relatives, colleagues, buddies or staff for performance of behaviour)* and* Change, or advise to change the social environment in order to facilitate performance of watned behaviour or create barriers to the unwanted behaviour (other than prompts/cues, rewards and punishment)
Any changes to electronic medical records (EMRs) were coded as *Restructuring physical environment (see above).* However, if forms were added to the EMR, that was coded as *Adding objects to the environment*.	*Adding objects to the environment*	Add object to the environment in order to facilitate the performance of the wanted behaviour

### Data analysis

Identified BCTs for both implementation and de-implementation interventions were counted and ranked according to frequency. Chi-squared (*χ*^2^) tests (Yates’ continuity correction for cells less than 5; and Fisher’s exact tests for cells equal to 0) were used to compare frequencies of BCTs between implementation and de-implementation interventions [27, 28], with significance (*p* value) adjusted for multiple comparison.

## Results

### Selection of intervention articles

The three reviews included 255 articles describing interventions to change health professionals’ behaviour. Screening of titles, abstract and summaries resulted in the exclusion of 63 articles (see Supplemental file #1 for list of excluded articles). Articles were excluded because either the behaviour change was not described as a change in frequency or the desired direction of the change was unclear.

### Inter-rater reliability

Inter-rater reliability of 26 double-screened articles was ‘substantial’ or ‘very good’ [25, 26] (*κ* = 0.839; 95% CI 0.626 to 1.000).

### Full-text screening and data extraction

**Fig. 1:**
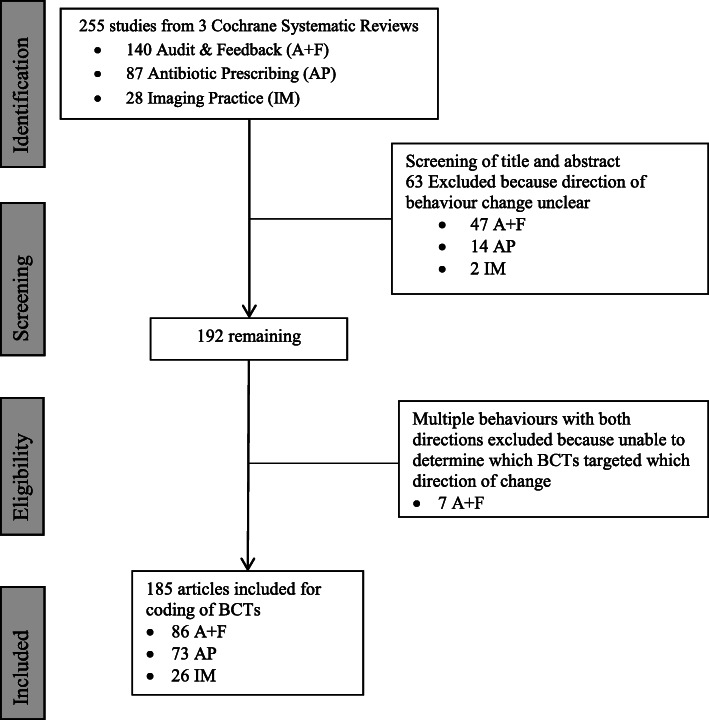
Flow diagram adapted from PRISMA to identify articles from three EPOC Systematic Reviews for BCT coding

Full-text screening and data extraction of the remaining 192 articles resulted in the exclusion of seven articles. These articles reported interventions designed to change multiple behaviours and, from the published intervention descriptions, it was unclear which behaviour change techniques were targeting which behaviours. Figure 1 presents the flowchart for the identification of articles to be coded for BCTs. Supplemental File #2 contains characteristics of the included studies.

Of the 185 articles, 84 described implementation interventions and 101 described de-implementation interventions (see Table 2). The majority of studies for both implementation and de-implementation targeted a single healthcare professional group (*n*=56 and *n*=55, respectively; e.g. primary care physicians, internists, dentists). The remaining interventions targeted multiple professional groups (e.g. physicians and nurses in a practice, physicians in various hospital departments) or included the entire hospital population in the intervention. For the most part, study designs were either cluster-randomised controlled trials (implementation *n* = 59; de-implementation *n* = 31) or randomised control trials (implementation *n* = 16; de-implementation *n* = 16). However, interrupted time series (implementation *n* = 5; de-implementation *n* = 52), controlled before-and-after (implementation *n* = 2; de-implementation *n* = 1), and clustered controlled trials (implementation *n* = 1; de-implementation *n* = 1), were also reported. In study descriptions that identified a control group (*n* = 123), only 20 implementation interventions and eight de-implementation interventions described the control condition. The remaining studies with control groups reported that the control group ‘did not receive an intervention’ or ‘received usual care’.

**Table 2:** Descriptive characteristics for articles included for BCT coding

Characteristics	Implementation interventions (*n* = 84)	De-implementation interventions (*n* = 101)
Systematic review		
Audit and feedback	60	26
Antibiotic prescribing	10	63
Image ordering	14	12
Target professionals		
Single physician group	56	55
Mixed professional group	30	22
Hospital population	8	24
Health professionals sample size		
<100	22	10
101-200	16	12
>200	14	9
Not reported/unclear	32	70
Study design		
Randomised controlled trial	16	16
Cluster randomised controlled trial	59	31
Interrupted time series	5	52
Controlled before-and-after	2	1
Cluster controlled trial	1	1
Control group		
Studies with BCTs	20	8

### Coding of intervention descriptions into behaviour change techniques

Agreement for BCT coding was 85% (AMP and HM) and 91% (AMP and KM). Sample BCT coding of an intervention description is provided in Supplemental file #3. Seven interventions descriptions could not be coded because there was not enough information to code [29–35].

In the remaining 178 articles, 25 of 93 possible BCTs (26%) were coded in de-implementation interventions and 29 (32%) were identified in implementation interventions. Table 3 provides a list of BCTs identified as well as definitions. Supplemental file #4 provides lists of BCTs coded in each implementation and de-implementation intervention descriptions.

**Table 3:** BCTs Identified in the study descriptions and definitions

BCTs identified	BCT definitions from Michie et al. [17]
Goal setting (behaviour)	Set or agree on a goal defined in terms of the behaviour to be achieved
Feedback on behaviour	Monitor and provide informative or evaluative feedback on performance of the behaviour (*e.g. form, frequency, duration, intensity*)
Monitoring of behaviour by others without feedback	Observe or record behaviour with the person’s knowledge as part of the behaviour change strategy
Behaviour substitution	Prompt substitution of the unwanted behaviour with a wanted or neutral behaviour
Restructuring social environment	Change, or advise to change the social environment in order to facilitate performance of the wanted behaviour or create barriers to the unwanted behaviour (other than prompts/cues, rewards and punishments)
Problem solving	Analyse, or prompt the person to analyse, factors influencing the behaviour and generate or select strategies that include overcoming barriers and/or increasing facilitators
Goal setting (outcome)	Set or agree on a goal defined in terms of a positive outcome of wanted behaviour
Action planning	Prompt detailed planning of performance of the behaviour (must include at least one of context, frequency, duration and intensity) context may be environmental (physical or social) or internal (physical, emotional or cognitive)
Review of behaviour goal(s)	Review behaviour goal(s) jointly with the person and consider modifying goals or behaviour change strategy in light of achievement. This may lead to resetting the same goal, a small change in that goal or setting a new goal instead of (or in addition to) the first, or no change
Discrepancy between current behaviour and goal	Draw attention to discrepancies between a person’s current behaviour (in terms for form, frequency duration, or intensity of that behaviour) and the person’s previously set of outcome goals, behavioural goals or action plans (goes beyond self-monitoring of behaviour)
Behavioural contract	Create a written specification of the behaviour to be performed, agreed on by the person and witnessed by another
Commitment	Ask the person to affirm or reaffirm statements indicating commitment to change the behaviour
Self-monitoring of behaviour	Establish a method for the person to monitor and record their behaviour(s) as part of the behaviour change strategy
Monitoring of behaviour by others without feedback	Observe or record outcomes of behaviour with the person’s knowledge as part of the behaviour change strategy
Feedback on outcome of behaviour	Monitor and provide feedback on the outcome of performance of the behaviour
Social support (unspecified)	Advise on, arrange or provide social support (*e.g. from friends, relatives, colleagues, ‘buddies’ or staff*) or non- contingent praise or reward for performance of the behaviour*.* It includes encouragement and counselling, but only when it is directed at the behaviour
Social support (practical)	Advise on, arrange or provide practical help (*e.g. from friends, relatives, colleagues, ‘buddies’ or staff*) or non-contingent praise or reward for performance of the behaviour*.* It includes encouragement and counselling, but only when it is directed at the behaviour
Instruction on how to perform the behaviour	Advise or agree on how to perform the behaviour (includes ‘Skills training’)
Information about health consequences	Provide information (e.g. written, verbal, visual) about health consequences of performing the behaviour
Information about social and environmental consequences	Provide information (e.g. written, verbal, visual) about social and environmental consequences of performing the behaviour. *Note: consequences can be for any target, not just the recipient(s) of the intervention*
Demonstration of the behaviour	Provide an observable sample of the performance of the behaviour, directly in person or indirectly, e.g. via film, pictures, for the person to aspire to or imitate.
Social comparison	Draw attention to others’ performance to allow comparison with the person’s own performance *Note: being in a group setting does not necessarily mean that social comparison is actually taking place*
Prompts and cues	Introduce or define environmental or social stimulus with the purpose of prompting or cueing the behaviour. The prompt or cue would normally occur at the time or place of performance
Behavioural practice/rehearsal	Prompt practice or rehearsal of the performance of the behaviour one or more times in a context or at the time when the performance may not be necessary, in order to increase habit or skill
Credible source	Present verbal or visual communication from a credible source in favour of or against the behaviour *Note: code this BCT if source generally agreed on as credible e.g. health professionals, celebrities or words used to indicate expertise or leader in field*
Pros and cons	Advise the person to identify and compare reasons for wanting (pros) and not wanting to (cons) change the behaviour (includes decisional balance)
Comparative imagining of future outcomes	Prompt or advise the imagining and comparing of future outcomes of changed versus unchanged behaviour
Material incentive (behaviour)	Inform that money, vouchers or other valued objects will be delivered if and only if the has been effort and/or progress in performing the behaviour (includes positive reinforcement)
Material reward (behaviour)	Arrange the delivery of money, vouchers or other valued objects if and only if the has been effort and/or progress in performing the behaviour
Non-specific incentive	Arrange the delivery or a reward if and only if there has been effort and /or progress in performing the behaviour.
Restructuring physical environment	Change, or advise to change the physical environment in order to facilitate performance of the wanted behaviour or create barriers to the unwanted behaviour (other than prompts/cues, rewards and punishments)
Adding objects to the environment	Add object to the environment in order to facilitate the performance or the behaviour. *Note: Provision of information* (*e.g. written, verbal, visual*) *in a booklet or leaflet is insufficient.*

### Frequency of BCTs in implementation versus de-implementation interventions

**Fig. 2:**
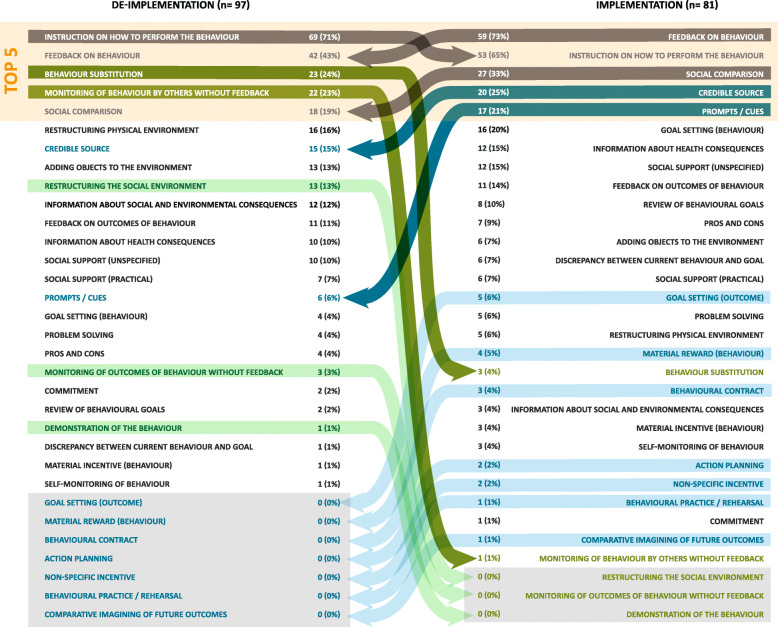
BCTs identified in implementation and de-implementation interventions ranked by frequency; grey arrows indicate BCTs present in top 5 for both implementation and de-implementation; green arrows indicate BCTs present more frequently in de-implementation (dark green—top 5 de-implementation; light green—BCT not present in implementation); blue arrows indicate BCTs present more frequently in implementation (dark blue—top 5 de-implementation; light blue—BCT not present in de-implementation)

**Fig. 3:**
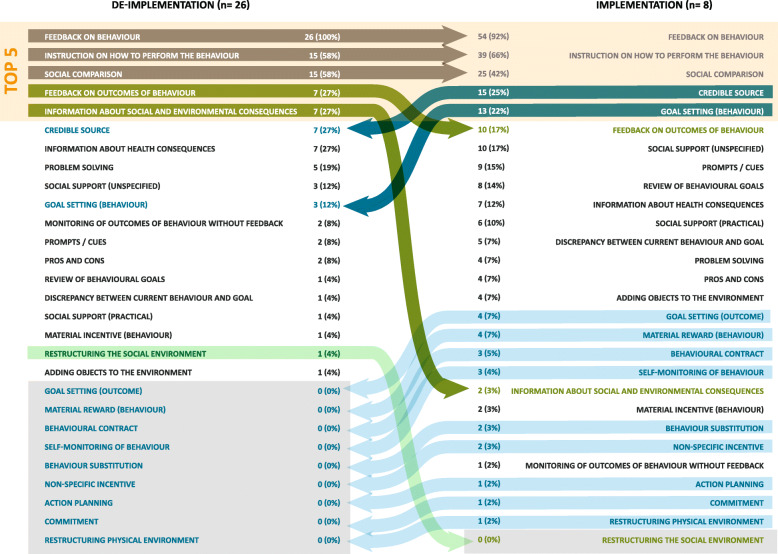
BCTs identified in audit and feedback implementation and de-implementation interventions ranked by frequency; grey arrows indicate BCTs present in top 5 for both implementation and de-implementation; green arrows indicate BCTs present more frequently in de-implementation (dark green—top 5 de-implementation; light green—BCT not present in implementation); blue arrows indicate BCTs present more frequently in implementation (dark blue—top 5 de-implementation; light blue—BCT not present in de-implementation)

**Fig. 4:**
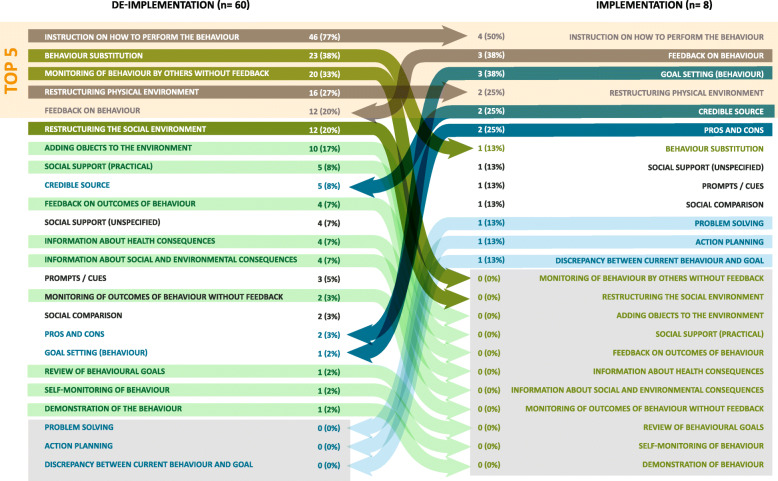
BCTs identified in antibiotic prescribing implementation and de-implementation interventions ranked by frequency; grey arrows indicate BCTs present in top 5 for both implementation and de-implementation; green arrows indicate BCTs present more frequently in de-implementation (dark green—top 5 de-implementation; light green—BCT not present in implementation); blue arrows indicate BCTs present more frequently in implementation (dark blue—top 5 de-implementation; light blue—BCT not present in de-implementation)

**Fig. 5:**
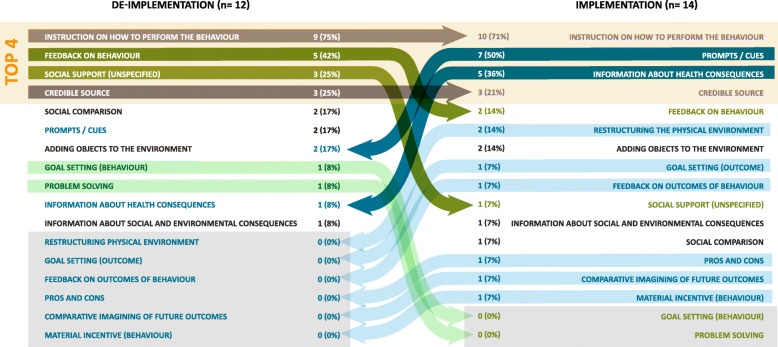
BCTs identified in image ordering implementation and de-implementation interventions ranked by frequency; grey arrows indicate BCTs present in top 5 for both implementation and de-implementation; green arrows indicate BCTs present more frequently in de-implementation (dark green—top 5 de-implementation; light green—BCT not present in implementation); blue arrows indicate BCTs present more frequently in implementation (dark blue—top 5 de-implementation; light blue—BCT not present in de-implementation)

Comparisons were made between implementation and de-implementation interventions across all studies (Fig. 2) and within each review (Figs. 3, 4, and 5 present the frequencies of BCTs identified in the implementation and de-implementation interventions in Audit and feedback, Antibiotic prescribing, and Image ordering reviews, respectively).

The most frequent BCTs in the implementation interventions were *Feedback on behaviour* (1st), *Instruction on how to perform the behaviour* (2nd), *Social comparison* (3rd), *Credible source* (4th), and *Prompts/cues* (5th). In the de-implementation interventions, the most frequent BCTs were *Instruction on (how) to perform the behaviour* (1st), *Feedback on behaviour* (2nd), *Behaviour substitution* (3rd), *Monitoring of behaviour by others without feedback* (4th) and *Social comparison* (5th). BCTs that were identified in implementation interventions and not in de-implementation were *Goal setting* *(outcome)* (*n* = 5), *Material reward (behaviour*) (*n* = 4), *Behavioural contract* (*n* =3), *Action planning* (*n*=2), *Non-specific incentive* (*n* = 2), *Behavioural practice/rehearsal* (*n* = 1) and *Comparative imagining of future outcomes* (*n* = 1). BCTs that were identified in de-implementation interventions and not in implementation were *Restructuring the social environment* (*n* = 13), *Monitoring of outcomes of behaviour without feedback* (*n* =3) and *Demonstration of the behaviour* (*n*=1).

#### Audit and feedback interventions

Twenty-seven BCTs were identified in implementation interventions and 19 were identified in de-implementation interventions (Fig. 3). The most frequent BCTs in the implementation interventions were *Feedback on behaviour* (1st), *Instruction on (how) to perform the behaviour* (2nd), *Social comparison* (3rd), *Credible source* (4th) and *Goal setting (behaviour)* (5th). In the de-implementation interventions, the most frequent BCTs were *Feedback on behaviour* (1st), *Instruction on (how) to perform the behaviour* (2nd), *Social comparison* (3rd), *Feedback on outcomes of behaviour* (4th) and *Information about social and environmental consequences* (5^th^).

#### Antibiotic prescribing interventions

Thirteen BCTs were identified in implementation interventions whilst 21 were identified in de-implementation interventions (Fig. 4). The most frequent BCTs in implementation interventions were *Instruction on (how) to perform the behaviour* (1st), *Feedback on behaviour* (2nd), *Goal setting* (behaviour) (2nd), *Restructuring physical environment* (3rd), *Credible source* (3rd) and *Pros and cons* (4th). In the de-implementation interventions, the most frequent BCTs were *Instruction on (how) to perform the behaviour* (1st), *Behaviour substitution* (2nd), *Monitoring of behaviour by others without feedback* (3rd), *Restructuring physical environment* (4th) and *Feedback on behaviour* (5th) and *Restructuring physical environment* (5th).

#### Image ordering interventions

Fifteen BCTs were identified in implementation interventions and 11 BCTs were identified in de-implementation interventions (Fig. 5). The most frequent BCTs identified in implementation interventions were *Instruction on (how) to perform the behaviour* (1st), *Prompts and cues* (2nd), *Information about health consequences* (3rd), and *Credible source* (4th). In the de-implementation interventions, the most frequent BCTs identified were *Instruction on (how) to perform the behaviour* (1st), *Feedback on behaviour* (2nd), *Social support* *(unspecified)* (3rd) and *Credible source* (4th).

**Fig. 6:**
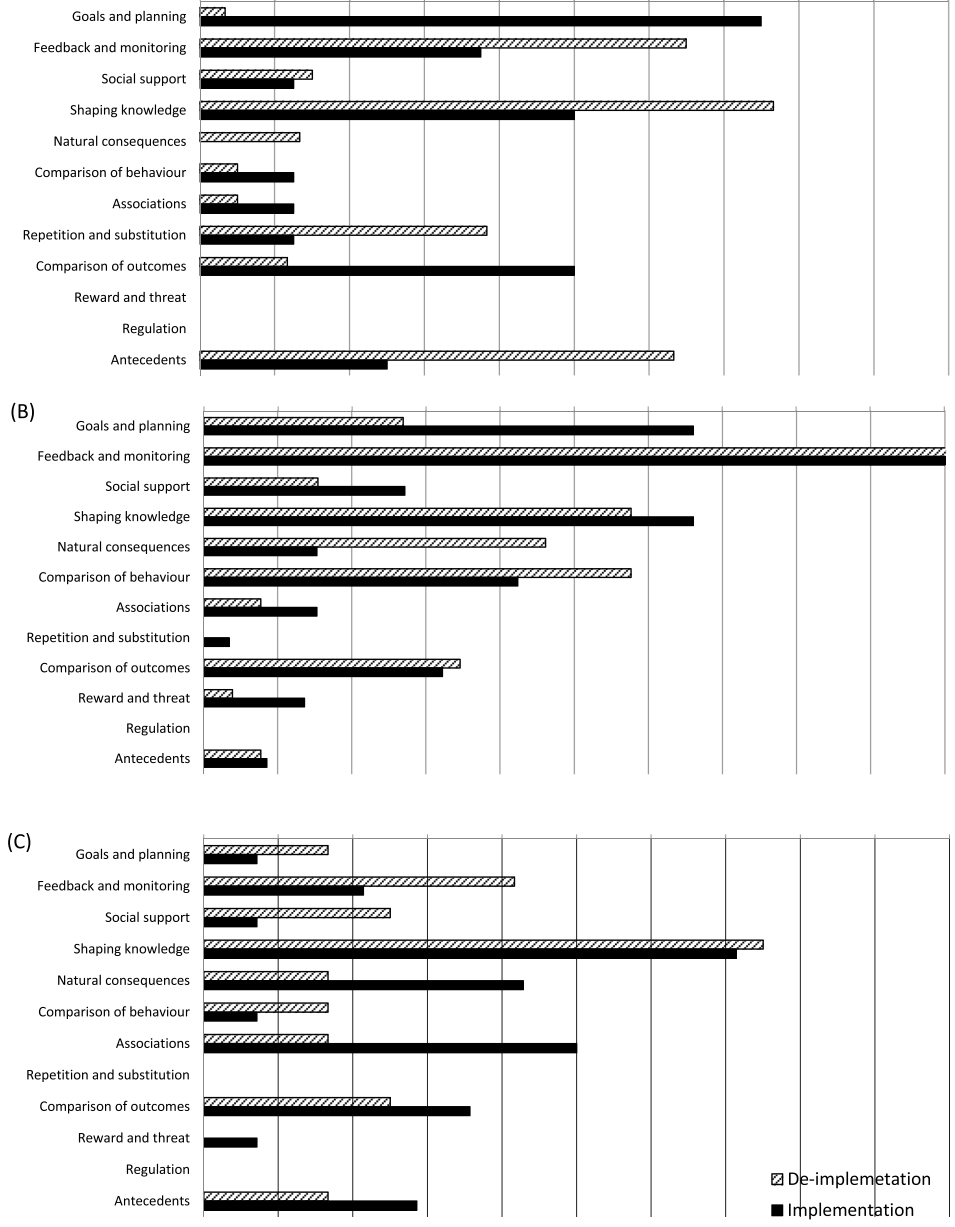
Comparison of BCTs in implementation and de-implementation interventions at the BCT grouping level for (**a**) Antibiotic prescribing, (**b**) Audit and feedback and (**c**) Image ordering systematic reviews

Comparison BCTs of implementation and de-implementation interventions at the level of taxonomy hierarchical grouping showed no grouping of BCTs was consistently reported more frequently in implementation or de-implementation interventions (see Fig. 6). No intervention descriptions contained BCTs within the groupings: *Regulation*, *Identity*, *Scheduled consequences*, *Self-belief* and *Covert learning*.

### Data analysis

Table 4 reports the significant associations between direction of change (implementation versus de-implementation) and the BCTs reported in intervention description (*p* <.0015 adjusted for 32 comparisons; applying the Yates’ continuity correction for cells less than 5 and Fisher’s exact test for cells equal to 0). Specifically, *Feedback on behaviour* was identified more frequently in implementation than de-implementation (*Χ*^*2*^(2, *n*=178) = 15.693, *p* = .000057). Three BCTs were identified more frequently in de-implementation than implementation; *Behaviour substitution* (*Χ*^*2*^(2, *n*=178) = 14.561, *p* = .0001; Yates’ continuity correction); *Monitoring of behaviour by others without feedback* (*Χ*^*2*^(2, *n*=178) = 16.187, *p* = .000057; Yates’ continuity correction); and *Restructuring social environment* (*p* = .000273; Fisher’s 2-sided exact test).

**Table 4:** Association between desired change in behaviour (implementation (*n*=81) and de-implementation (*n*=97)) and BCT present

BCT	Desired change in behaviour	BCT identified		Value	Significance value ^**+**^
		Present	Absent		
**Behaviour substitution**	Implementation	3	78	**12.607**	<.0005*^b^
	**De-implementation**	**23**	74		
**Feedback on behaviour**	**Implementation**	**59**	22	**15.693**	<.0001*^a^
	De-implementation	42	55		
**Monitoring of behaviour by others without feedback**	Implementation	1	80	**16.187**	<.0001*^b^
	**De-implementation**	**22**	75		
**Restructuring social environment**	Implementation	0	81	**--**	<.0005*^c^
	**De-implementation**	**13**	84		
Goal setting (behaviour)	Implementation	16	65	9.301	.002^b^
	De-implementation	4	93		
Problem solving	Implementation	5	75	0.077	.781^b^
	De-implementation	4	93		
Goal setting (outcome)	Implementation	5	76	--	.018^c^
	De-implementation	0	97		
Action planning	Implementation	2	79	--	.206^c^
	De-implementation	0	97		
Review of behaviour goal(s)	Implementation	8	73	3.717	.054^b^
	De-implementation	2	95		
Discrepancy between current behaviour and goal	Implementation	6	75	3.213	.073^b^
	De-implementation	1	96		
Behavioural contract	Implementation	3	78	--	.092^c^
	De-implementation	0	97		
Commitment	Implementation	1	80	--	.455^c^
	De-implementation	0	97		
Self-monitoring of behaviour	Implementation	3	78	.477	.490^b^
	De-implementation	1	96		
Monitoring of behaviour by others without feedback	Implementation	0	81	--	.501^c^
	De-implementation	2	95		
Feedback on outcome of behaviour	Implementation	11	70	.204	.651^a^
	De-implementation	11	86		
Social support (unspecified)	Implementation	12	12	.827	.363^a^
	De-implementation	10	87		
Social support (practical)	Implementation	6	75	.105	.746^a^
	De-implementation	6	91		
Instruction on how to perform the behaviour	Implementation	53	28	.666	.415^a^
	De-implementation	69	28		
Information about health consequences	Implementation	12	69	.827	.363^a^
	De-implementation	10	87		
Information about social and environmental consequences	Implementation	3	78	3.247	.072^b^
	De-implementation	12	85		
Demonstration of the behaviour	Implementation	0	81	--	1.00^c^
	De-implementation	1	96		
Social comparison	Implementation	27	54	5.102	.024^a^
	De-implementation	18	79		
Prompts and cues	Implementation	17	64	8.595	.003^a^
	De-implementation	6	91		
Behavioural practice/rehearsal	Implementation	1	80	--	.455^c^
	De-implementation	0	97		
Credible source	Implementation	20	61	2.379	.123^a^
	De-implementation	15	82		
Pros and cons	Implementation	7	74	.873	.350^b^
	De-implementation	4	93		
Comparative imagining of future outcomes	Implementation	1	80	--	.455^c^
	De-implementation	0	97		
Material incentive (behaviour)	Implementation	3	78	.477	.490^b^
	De-implementation	1	96		
Material reward (behaviour)	Implementation	4	77	--	.041^c^
	De-implementation	0	97		
Non-specific incentive	Implementation	2	79	--	.206^c^
	De-implementation	0	97		
Restructuring physical environment	Implementation	5	81	4.519	.034^a^
	De-implementation	16	76		
Adding objects to the environment	Implementation	6	75	1.664	.197^a^
	De-implementation	13	84		

^+^Significance value adjusted for 32 comparisons (Bonferroni; *p*< .0015)

^a^Pearson’s chi-square

^b^Yates’ continuity correction for cells less than 5

^c^Fisher’s exact test for cells with 0 count

## Discussion

This study investigated whether implementation and de-implementation interventions as described in three purposively sampled Cochrane reviews contain different BCTs. Three of the BCTs reported more frequently in de-implementation interventions (*Behaviour substitution, Restructuring social environment* and *Monitoring of behaviour by others without feedback*) if associated with effective de-implementation interventions may help refine methods (and theory) for the design of de-implementation interventions.

*Behaviour substitution* is a technique that may be helpful in some de-implementation intervention designs. When designing theory-informed interventions, substitute behaviours were often introduced and the theories applied were used to increase the frequency of the substitute behaviour in order to de-implement the undesired or incompatible behaviour [12, 13]. When investigating determinants of behaviours in which the behaviour under investigation was a behaviour the HCP should avoid doing, researchers tended to frame the behaviour as ‘managing [clinical patient] without doing “x”’ [36–38]. Whilst permitting the respondents to reflect on the management of the patient rather than the specific behaviour under investigation, the framing of the question implies that the respondents could be doing something else.

One of the main benefits of using *Behaviour substitution* to de-implement behaviour is that it permits the healthcare professional (HCP) to focus on doing something, rather than doing nothing for the care of their patients. However, there are also potential challenges to using *Behaviour substitution*. Currently, we do not have methods for selecting appropriate substitute behaviours and the rationale for selection of substitute behaviour is rarely reported [12]. Researchers may have resorted to intuitive or pragmatic ideas within each context, resulting in no cumulative learning on how best to identify the behaviour to promote in *Behaviour substitution*. There is also the challenge of what to do in clinical contexts where there is no sensible substitute behaviour. This is probably less common in healthcare because, in the absence of performing the undesired behaviour, HCPs will likely decide to monitor the patient more closely or use strategies to address patient concerns. Example substitute behaviours in these cases are (a) for reducing unnecessary blood transfusions, the clinician continues monitoring the patient and orders additional tests [37]; (b) for deciding not to order an X-ray, the physician discusses their reasoning with the patient who has acute low back pain [39, 40]; (c) for deciding not to prescribe antibiotics in respiratory tract infections, the clinician could provide a viral prescription to the patients with symptom management strategies [41]. Further investigation is needed to clarify when *Behaviour substitution* would be effective (for what clinical behaviours and in what contexts).

*Restructuring social environment* may also be a candidate BCT for refining methods for de-implementation. For example, in many of the interventions for de-implementing antibiotic prescribing, HCPs were required to get approval from a senior or secondary clinician, or directly from the pharmacy, when requesting an antibiotic to be de-implemented. Seeking another clinician’s approval may be just enough of an inconvenience to deter the HCP from requesting the low-value care and the ease of order/prescribing is no longer there due to the added actions required. However, there may be unintended consequences of using this technique. Clinicians may perceive that their autonomy and clinical expertise is being challenged and there may be resistance. Additionally, applying this BCT would require additional labour resources that may not be available. Personnel would have to be willing to take on the added role and be available to review the requests. Identifying available resources to support application of this technique as well as addressing potential unintended consequences may be key factors its effectiveness and requires further study.

*Monitoring of behaviour by others without feedback* was reported more frequently in de-implementation than implementation. It is unclear why this is the case. One possibility is that de-implementation interventions historically have focused on quality improvement or cost saving measures and this BCT may have been applied as an organisational measure to document reduction in wasteful practice and cost as well as restrict clinicians’ practice behaviours. In addition, this BCT may work in the same manner as *Restructuring social environment* because clinicians would require other individuals to record and perhaps evaluate their practice behaviour. However, the unintended consequences of HCPs perceiving their autonomy and clinical expertise being challenged resulting in resistance, may be a concern when using this technique.

### Strengths and limitations

This study was the first to apply the BCT taxonomy (v1) to compare implementation and de-implementation interventions that support HCP behaviour change. The taxonomy provided a relatively precise, ‘shared language’ [17] to describe a diverse range of implementation and de-implementation interventions. However, there were limitations to the current study.

It is important to note that our findings do not imply the effectiveness of the BCTs for implementation or de-implementation, rather simply that they were more frequently used and suggest that researchers who designed the interventions may have ‘implicit theories’ [42, 43] about different BCTs required for de-implementation and implementation. Whilst we would have liked to include effectiveness, we were unable to undertake any formal meta-analysis because studies across the three reviews were not sufficiently similar in terms of interventions evaluated and outcomes measured for the findings to be meaningful. Additionally, effectiveness reported in the Cochrane reviews included in this study, could not be attributed to a single BCT (whether used of de-implementation or implementation) but to the cluster of BCTs identified in the intervention. Therefore, we believe it would be premature to include reported effectiveness of these interventions without being able to attribute effectiveness to the specific BCT, as well as before fully understanding the intervention components and the fidelity of intervention delivery.

We found that 32 of the 93 possible BCTs were used in the interventions included in this study. Although it is possible that not all 93 BCTs would be appropriate in the settings investigated, this does suggest that there may be additional potentially effective techniques that researchers have not yet explored. Additionally, as we have indicated in the introduction, the BCT taxonomy is one of a number of taxonomies that could have been used to identify intervention strategies. We chose to use the BCT taxonomy for its granularity and specificity of techniques but recognise that, whilst a comprehensive list, it is not a complete list of all possible techniques and may omit techniques relevant to identify difference between implementation and de-implementation. The BCT taxonomy is currently in its first version and opportunities exist to add to or refine the taxonomy as new evidence and research emerges.

Whilst the number of studies in both implementation (*n*=81) and de-implementation (*n*=97) were similar, the number of studies in the reviews were skewed to either implementation or de-implementation. For example, the Audit and Feedback review included twice as many implementation studies as de-implementation studies (*n*=59, *n*=26 respectively). In the Antibiotic Prescribing review, the number of implementation studies was considerably smaller than de-implementation studies (*n*=8, *n*=60 respectively). BCTs in implementation interventions were found in a large number of audit and feedback studies and BCTs in de-implementation interventions were found in the majority of antibiotic prescribing studies. Whilst we have identified differences in the BCTs used for implementation and de-implementation interventions, these findings are limited to three Cochrane reviews and require replication for a wider range of clinical behaviours.

In addition, multiple statistical comparisons reduce power and therefore require that the significance criterion be adjusted to control for type 1 error. For this study, we applied a conservative approach and adjusted significance criterion to *p*<.00015 for the 32 comparisons. It could be argued that the significant criterion may have been too conservative and a number of comparisons that were not statistically significant (.05<*p*>.00015) would be significant without the adjusted significant criterion, including *Goal setting* (behaviour), *Goal setting* (outcome), *Social comparison*, *Prompts and cues and Material reward* identified more frequently in implementation and *Restructuring physical environment* identified more frequently in de-implementation. However, our conservative approach strengthens the confidence with which inferences can be made about the BCTs that distinguish between increasing and decreasing behaviour frequency, indicating a robust level of evidence that interventions contain some different techniques for implementation and de-implementation.

### Implications for future work

As previously mentioned, we did not investigate effectiveness of identified BCTs. This is clearly a next logical step in understanding the differences between the processes of implementation and de-implementation. A recent study has proposed links from groups of commonly used BCTs in interventions to behaviour change theories, suggesting that there is a possible common theorising about how BCTs may work together which is grounded in behaviour change theories [44]. Our findings suggest that common theorising (even if implicit) may also exist in selecting different BCTs for de-implementation than implementation. Future work should include linking the BCTs used for de-implementation and their effectiveness to behaviour theories, which could guide further theoretical understanding about de-implementation.

Additionally, there is considerable opportunity to utilise the BCT Taxonomy (v1) [17] in other systematic reviews to determine if our findings are consistent with other comparisons of implementation and de-implementation interventions. Applying the same methods to intervention descriptions from other reviews, may identify whether these underreported BCTs were used more frequently or if they are consistently underused regardless of nature of the clinical behaviour or the clinical context. Because of the limited range of BCTs reported in the intervention descriptions of 187 published articles, there is opportunity to develop novel interventions that contain BCTs for the groups not reported and measure the effectiveness of those interventions to de-implement low-value care. It is also possible that the BCTs reported may not have been appropriate for the interventions because the majority of BCTs originated in context of clinical psychology, in which one-on-one interventions are delivered to people who have already acknowledged a need to change. Delivering the same BCTs in HCP behaviour change interventions may not be appropriate because of the different contexts and populations. Future research could develop novel interventions that contain BCTs for the underused BCT groupings. Investigating the application and effectiveness of less utilised BCTs may prove informative in interventions for de-implementation.

## Conclusion

Policy interest in de-implementation has raised the question of whether the approaches to implement and de-implement are similar or different [4–6, 9, 11]. Implementation research has not yet addressed this question. This study has found that BCTs *Behaviour substitution, Restructuring social environment* and *Monitoring of behaviour by others without feedback* are frequent techniques for de-implementing low-value behaviours, and *Feedback on behaviour* was identified more frequently for implementing high-value care. Whilst there were some significant associations between BCTs reported in interventions and the direction of desired behaviour change (i.e. more or less, or implementation and de-implementation, respectively), there was also variation in BCTs included in these two kinds of interventions. These findings require replication for a wider range of clinical behaviours. The continued accumulation of knowledge and evidence into differences between the processes of implementation and de-implementation will serve to better inform researchers and, subsequently, improve methods for designing de-implementation interventions.

## Supplementary Information

**Additional file 1.** Supplemental File 1 List of intervention articles excluded from BCT coding

**Additional file 2.** Supplemental File 2 Characteristics of included intervention articles for BCT coding

**Additional file 3.** Supplemental File 3 Sample of BCT coding of intervention description

**Additional file 4.** Supplemental File 4 BCTs identified in intervention descriptions

## Data Availability

The datasets used and/or analysed during the current study are available from the corresponding author on reasonable request.

## Abbreviations

BCT: Behaviour Change Techniques
HCP: Healthcare Professional
EPOC: Effective Practice and Organization of Care
