# Supplementary material for: Changing behaviour, ‘more or less’: do implementation and de-implementation interventions include different behaviour change techniques?
Source: Implement Sci. 2021 Feb 25;16:20. doi: 10.1186/s13012-021-01089-0 (PMC7905859; doi:10.1186/s13012-021-01089-0)
Supplement: Supplementary file 1 — Additional file 1. Supplemental File 1 List of intervention articles excluded from BCT coding [file 13012_2021_1089_MOESM1_ESM.docx]

Supplemental File #1 List of intervention articles excluded from BCT coding

| **Authors** | **Systematic review** | **Design** | **Target Behaviour** | **Reason for exclusion** |
| --- | --- | --- | --- | --- |
| Bahrami et al., 2004 | A+F | cRCT | Adhere to the SIGN guideline for management of impacted and un-erupted third molars | Direction unclear |
| Baker et al, 2003 | A+F | cRCT | Improve test ordering (can be both increasing and Decreasing depending on the pt.) | Direction unclear |
| Baker et al., 1997 | A+F | cRCT | Improve management of patients taking benzodiazepines long term (guideline compliance) | Behaviour change is not described as a frequency change |
| Blais et al., 2008 | A+F | RCT | Improve use of medication to treat asthma | Behaviour change is not described as a frequency change |
| Boekeloo et al., 1990 | A+F | cRCT | Increase adherence to cholesterol management opportunities | Behaviour change is not described as a frequency change |
| Borer et al., 2004 | AB | RCT | Unclear (provide appropriate therapy) | Direction unclear |
| Borgiel et al., 1999 | A+F | RCT | Improve quality of care by GPs | Behaviour change is not described as a frequency change |
| Bouza et al., 2004 | AB | RCT | Unclear (provide adequate therapy) | Behaviour change is not described as a frequency change |
| Bregnhoj et al., 2009 | A+F | cRCT | Improve prescribing in older adults | Behaviour change is not described as a frequency change (appropriateness based on the MAI incorporates explicit criteria and uses implicit instructions. |
| Bruins et al., 2005 | AB | RCT | Improve clinical outcomes (undefined) | Not a change in frequency (appropriate use) |
| Buntinx et al., 1993 | A+F | cRCT | Improve quality of cervical smears (technique of smear) | Behaviour change is not described as a frequency change (technique of smear) |
| Burton et al., 1991 | AB | RCT | Use Bayesian calculation for dosage | Not a change in frequency (dose adjustment) |
| Charrier et al., 2008 | A+F | RCT | Adhere to protocols for preventing pressure lesions and managing peripheral and central venous catheters | Direction unclear (+30 checklist for number of behaviours) |
| Cheater et al., 2006 | A+F | cRCT | Comply with review criteria for assessment and management of urinary incontinence and impact on psychological and social well-being and symptoms | Behaviour change is not described as a frequency change (compliance to assessment and mgmt. of incontinence) |
| Claes et al., 2005 | A+F | cRCT | Improve quality of oral anticoagulation therapy in GP's | Behaviour change is not described as a frequency change (he quality of anticoagulation management, defined as the proportion of time that INR-values were within target range) |
| Curran et al., 2008 | A+F | cRCT | Decrease MRSA infections | Behaviour change is not described as a frequency change (i.e. change in outcome) |
| De Almeida Neto et al., 2000 | A+F | cRCT | Improved pharmacist participation in counselling clients for pharmacist only (non-prescription) prescriptions | Behaviour change is not described as a frequency change |
| Dean et al., 2001 | AB | cRCT | Change antibiotic timing and selection (unclear in abstract) (compliance with guidelines to reduce community acquired pneumonia) | Behaviour change is not described as a frequency change (implement guidelines) measure patient related - length of stay. 30-day mortality) |
| Dempsey et al., 1995 | AB | ITS | Improve clinical management of patients | Behaviour change is not described as a frequency change (patient quality outcomes and change in dosage of antibiotic and selection) |
| Eltayeb et al., 2005 | A+F | RCT | Improve prescribing for sexual transmitted diseases | Behaviour change is not described as a frequency change (unclear) |
| Filardo et al., 2009 | A+F | cRCT | Manage congestive heart failure and pneumonia care. | Direction of change unclear - composite of quality improvement study - outcome measure composite score of management |
| Gullion et al., 1998 | A+F | cRCT | Manage hypertension and blood pressure | Behaviour change is not described as a frequency change |
| Gums et al., 1999 | AB | cRCT | Improve suboptimal intravenous antibiotics | Behaviour change is not described as a frequency change (dosage and selection) -"optimal antibiotic choices and dosages" |
| Heller et al., 2001 | A+F | RCT | Manage unstable angina | Behaviour change is not described as a frequency change. |
| Hemminiki et al., 1992 | A+F | RCT | Improve quality of care | Unclear whether change in behaviour was a change in frequency. Quality of care. |
| Hendryx et al., 1998 | A+F | cRCT | Improve quality of care (processes, resources, outcomes) | Unclear whether change in behaviour is change in frequency (44 indicators) |
| Herrin et al., 2006 | A+F | cRCT | Improve diabetes care | Unclear whether change in behaviour is a change in frequency. |
| Lagerlov et al., 2000 | A+F | RCT | Improve appropriate (direction unclear) prescribing for asthma and urinary tract infections | Direction of change unclear. The difference in proportions of acceptably and unacceptably treated asthma patients before and after the intervention was calculated within the asthma and control (UTI) groups. |
| Landgren et al., 1988 | AB | CBA | Prescribe antibiotics appropriately based on duration and timing (no direction given) | Behaviour change is not described as a frequency change (duration of prophylaxis) |
| Linn et al., 1980 | A+F | cRCT | Improve emergency room burn care | Change unclear as a change in frequency and direction unclear. |
| Marton et al., 1985 | A+F | cRCT | Modify physician use of the laboratory | Change unclear as a change in frequency and direction unclear. |
| McAlister et al., 1986 | A+F | cRCT | Improve management of hypertension by GP's | Behaviour change is not described as a frequency change, Not sure what they are measuring. |
| McClellan et al., 2004 | A+F | RCT | Adhere to haemodialysis care guidelines | Direction of change frequency unclear - improvement of adequacy of care |
| Mol et al., 2005 | AB | ITS | Improve suboptimal prescribing of ciprofloxacin and co-amoxiclav. | change is not described as a change in frequency |
| Norton et al., 1985 | A+F | cRCT | Record cystitis and vaginitis in patient chart | change is not described as a change in frequency |
| Phillips et al., 2005 | A+F | cRCT | Manage diabetes | Unclear as to the behaviour being targeted (improving HBA1c levels in patients but not sure what behaviour MDs performed) |
| Raasch et al., 2000 | A+F | cRCT | Diagnose and manage suspicious skin lesions | Correct diagnosis behaviour not described as a frequency change |
| Rantz et al., 2001 | A+F | cRCT | Improve clinical practice in nursing | Change in direction not a change in frequency (behaviours unclear) |
| Rask et al., 2001 | A+F | cRCT | Adhere to guidelines re preventative services for diabetes | Change unclear as a change in frequency and direction unclear. |
| Robling et al., 2002 | IM/ A+F | RCT | Improve imaging practice according to guidelines | change is not a change in direction (don't know what the problem is) |
| Ruangkanchanasetr et al., 1993 | A+F | cRCT | Utilize laboratory investigations | Change unclear as a change in frequency and direction unclear. |
| Schectman et al., 2003 | IM | cRCT | Improve guideline adherence for acute low back pain (guideline compliance- behaviour described as 'general management of a problem') | change is not specific to one direction. Not a change in frequency. |
| Schneider et al., 2008 | A+F | cRCT | Improve asthma care (multiple guidelines) | direction of change unclear; not necessarily a change in frequency. |
| Senn et al., 2004 | AB |  | Improve appropriate antibiotic use | Change is not a change in frequency - it's a modification in duration at reassessment. |
| Sinclair et al., 1982 | A+F | cRCT | Implement a quality assurance model in children’s mental health | Change is unclear whether it was a change in frequency |
| Smith et al., 1998 (1995) | A+F | cRCT | Adhere to guidelines for sedative hypnotic medication | Quality of information given to parents for genetic screening |
| Socolar et al., 1998 | A+F | cRCT | Improve chart documentation and knowledge of physicians doing evaluations for child sexual abuse | Quality of chart documentation/ not a change in frequency |
| Stevenson et al., 1988 | AB | cRCT | Improve antibiotic prescribing (decrease cost) | Change is unclear whether it was a change in frequency |
| Svetkey et al., 2009 | A+F | RCT | Improve adherence to hypertension treatment guidelines | Unclear behaviours (adherence to guidelines doesn't report what behaviours the guidelines include) |
| Toltiz et al., 2002 | AB | cRCT | Follow rotation (gentamicin, piperacillin tazobactam, and ceftazidime) protocol to decrease antibiotic resistant Bacilli | change in behaviour is not a change in frequency |
| Trenholme et al., 1989 | AB | cRCT | Follow recommendations (compliance in Recommendations) | Change in behaviour is not specific change in direction. Depends on recommendation. |
| van den Hombergh et al., 1999 | A+F | ITS | Improve practice management in general | Change in behaviour not reported as a charge in frequency (208 quality indicators grouped in chapters and dimensions) |
| van der Weijden et al., 1999 | A+F | cRCT | Adhere to a cholesterol guideline | Unclear description of behaviour, adhering to cholesterol guidelines |
| Vingerhoets et al., 2001 | A+F | cRCT | GP care that results in improved patient evaluation of care | behaviour not reported as change in frequency. patient satisfaction with GP care |
| Walker, 1998 | AB | RCT | Change the Duration of patient’s drug and route of administration | Unable to retrieve article |
| Ward et al., 1996 | A+F | CCT | Manage Type 2 diabetes | Unclear behaviours |
| Weitzman et al., 2009 | A+F | RCT | Improve diabetes care (glycaemic, lipid, and blood pressure control) | Patient outcome measures (lower level of HBA1c, LDL and SBP measures) behaviour not a change in frequency |
| Ziemer et al., 2006 | A+F | cRCT | Improve provider adherence to guidelines to improve glycaemic control | Behaviour non-specific not described as a change in frequency. |
