# Supplementary material for: Changing behaviour, ‘more or less’: do implementation and de-implementation interventions include different behaviour change techniques?
Source: Implement Sci. 2021 Feb 25;16:20. doi: 10.1186/s13012-021-01089-0 (PMC7905859; doi:10.1186/s13012-021-01089-0)
Supplement: Supplementary file 2 — Additional file 2. Supplemental File 2 Characteristics of included intervention articles for BCT coding [file 13012_2021_1089_MOESM2_ESM.docx]

Supplemental File #2: Characteristics of included intervention articles for BCT coding

| **Authors** | **EPOC review** | **Direction of change** | **Target change** | **How change was measured** | **Target professionals** | **Study Design** |
| --- | --- | --- | --- | --- | --- | --- |
| Abramowitz 1982 | AB | Decrease | Reduce cost of antibiotic prescribing | Total cost of all antibiotic prescribed | All Clinicians responsible for Prescribing Antibiotics in hospital in US | ITS |
| Adachi 1997 | AB | Decrease | Limit use of Vancomycin to appropriate usage | Use of order sheet and Vancomycin prescribing | Physicians in US hospital responsible for ordering antibiotics | ITS |
| Anderson 1994 | A+F | Increase | Increase prescription for prophylaxis for venous thromboembolism | Patients were judged to have received adequate prophylaxis if one or more of (low- dose subcutaneous heparin, warfarin, intermittent pneumatic calf compression, or placement of an inferior vena caval filter or clip) methods were continued for at least 24 hours. | Physicians in 10 hospitals in the US | cRCT |
| Ansari 2003 | AB | Decrease | Reduce inappropriate use of Alert antibiotics | Use of alert antibiotics | physicians in one hospital in the UK excluding haematology and paediatrics | ITS |
| Avorn 1988 | AB | Decrease | Reduce antibiotic prescribing | Percentage of patients with kinetically incorrect dosing of cefazolin | Hospital Physicians | ITS |
| Awad 2006 | A+F | Decrease | Decrease inappropriate antibiotic prescribing | Antibiotic prescribing | Family physicians/ GP | cRCT |
| Bailey 1997 | AB | Decrease | Discontinue IV antibiotic | Duration of IV antibiotic | Physicians in two hospitals in the US | RCT |
| Baker 1987 | IM | Decrease | Reduce unnecessary lumbosacral radiography | Number of lumbosacral spine radiographies | Physicians in trauma centre in New york city | ITS |
| Baker 2003 | A+F | Increase | Improve care for adults with asthma and angina (number of behaviours) | The number of pathology tests ordered by general practices. | Family physicians/ GP | cRCT |
| Balas 1998 | A+F | Increase | Increase consideration of peritoneal dialysis as the preferred method | The number of patients allocated to peritoneal dialysis | Physicians in dialysis clinics. | cRCT |
| Barlow 2007 | AB | Increase | Improve antibiotic prescribing for patients within 4hr of admission | Appropriate antibiotics within 4 h of admission | Physicians at Acute & Emergency department | ITS |
| Batty 2001 | A+F | Decrease | Increase appropriate use of benzodiazepines in older adults | Observed and appropriate prescribing of benzodiazepines | Physicians, pharmacists and nurses involved in the care of elderly patients. | cRCT |
| Beck 2005 | A+F | Increase | Increase beta blocker prescribing | The proportion of elderly survivors of AMI at each study hospital who filled a prescription for a beta blocker within 30 days after discharge. | Internists | cRCT |
| Belliveau 1996 | AB | Decrease | Reduce vancomycin | Amount of vancomycin used | All physicians at one teaching hospital | ITS |
| Bentz 2007 | A+F | Increase | Increase rate of referral to a state-level tobacco quit-line | EHR-documented rates of advising, assessing, and assisting | Physicians from 19 primary care clinics | cRCT |
| Berild 2002 | AB | Decrease | Reduce the antibiotic consumption | Total antibiotic usage and usage of 5 specific groups | Paediatricians in hospital | ITS |
| Berman 1998 | A+F | Decrease | Reduce costs of drugs and supplies used by anaesthesiologists | Costs for carotid endarterectomies and for lumbar decompressions; use rates for propofol and etomidate and for patient warming devices. | Anaesthesiology residents | RCT |
| Bonevski 1999 | A+F | Increase | Increase rates of 3 screening behaviours (cholesterol, blood pressure, cervical screening) | Rates for cholesterol, blood pressure, cervical screening, and identify 3 risk behaviours (smoking, alcohol, benzodiazepines) | General practitioners | cRCT |
| Boyd 2002 | IM | Increase | Increase BMD Screening | Telephone surveyors asked patients about Screening | Primary care physicians | cRCT |
| Bradley 1999 | AB | Decrease | Decrease antibiotic use | Antibiotic usage | Physicians on a haematology ward | ITS |
| Brady 1988 | A+F | Increase | Increase influenza vaccination and breast cancer screening | Rates of influenza vaccination; rate of mammography ordering | Internists | cRCT |
| Brown 1994 | A+F | Increase | Increase number of patients with notation in chart | Percentage of records containing at least one periodontal diagnostic notation; percentage of records with at least one preventive notation and one treatment item | Dentists | cRCT |
| Buffington 1991 | A+F | Increase | Increase flu immunization | Influenza immunization rates of elderly patients seen in private physicians' offices | 45 physicians in 13 private practice groups agreed to participate. | cRCT |
| Bunz 1990 | AB | Decrease | Extend dosage interval of antibiotic prescription | Number of prescriptions | Physicians | ITS, UBA |
| Calil 2001 | AB | Decrease | Reduce inappropriate antibiotic prescribing | The incidence and prevalence of multi-resistant E cloacae | Health professional in one hospital neonatal unit. | ITS |
| Carling 2003 | AB | Decrease | Reduce antibiotic use | Use of antibiotics | Health professional in a hospital | ITS |
| Charbonneau 2006 | AB | Decrease | Decrease fluoroquinolone | Defined daily doses (DDDs) per 1000 bed-days of fluoroquinolone use | All antibiotic prescribers, including residents and senior physicians in a university hospital in France | ITS |
| Chassin 1986 | A+F | Decrease | Reduce inappropriate use of X-ray pelvimetry | Rate of pelvimetry | 1483 physicians at 120 hospitals in six states. | cRCT |
| Christ-Crain 2004 | AB | Decrease | Reduce antibiotic use in lower respiratory tract infections | Use of antibiotics | Physicians | cRCT |
| Christ-Crain 2006 | AB | Decrease | Reduce antibiotic use in community-acquired pneumonia. | Total antibiotic use (i.e., antibiotic prescription [percentage] | Physicians | RCT |
| Chu 2003 | AB | Increase | Improve the management of pneumonia | Use of antibiotics | Health professionals | CBA |
| Climo 1998 | AB | Decrease | Reduce the use of clindamycin | Use of antibiotics | Physicians | ITS |
| Cline 2007 | A+F | Increase | Increase referral rates | Hypertension specific referrals in discharge documentation for patients seen after an episode of hypertension, defined as a presenting blood pressure of 160/100 mm Hg or higher | ER Physicians | cRCT |
| Cohen 1982 | A+F | Decrease | Decrease physician test usage | Tests usage X-ray and lab tests | Physicians in hospital | cRCT |
| Curtis 2005 | A+F | Increase | Increase testing of CBC, Creatine, and use of cytoprotective agents. | The proportion patients with an increased risk for NSAID GI toxicity who had at least 1 CBC test performed); (2) the proportion of the physician’s patients with an increased risk for NSAID renal toxicity who had at least 1 creatinine test performed; and (3) the proportion of users of traditional NSAIDs with NSAID-related GI risk receiving a cytoprotective agent | Family physicians/ GP/ internists | cRCT |
| Curtis 2007 | IM | Increase | Increase osteoporosis medication prescription | Proportion of the long-term glucocorticoid users of each physician in the 1-year following the intervention who received prescription osteoporosis medication | Physicians treating long-term Glucocorticoid patients | cRCT |
| Curtis 2007a | A+F | Increase | Increase bone mineral density (BMD) testing | Proportion of the long-term glucocorticoid users of each physician in the 1-year following the intervention who underwent BMD testing | Physicians treating long-term Glucocorticoid patients | cRCT |
| De Champs 1994 | AB | Decrease | Use amikacin instead of gentamicin | Rate of amikacin use | Clinicians on neonatal/paediatric ward | ITS |
| De Man 2000 | AB | Decrease | Change from one group of antibiotics to another | Use over number of days. | Clinicians on two neonatal wards | cCCT |
| Dey 2004 | IM | Decrease | Improve management of low back pain | Referral rates within 3 mos.; number of prescribed opioids and relaxants; number of referrals to secondary care; number of referrals to physio or education program. | Primary care physicians | cRCT |
| Dranitisaris 2001 | AB | Decrease | Decrease inappropriate use of cefotaxime according to hospital guidelines | Percentage of prescribing consistent with guidelines. | Physicians managing patients with infections requiring IV cefotaxime | RCT |
| Eccles 2001 | IM /A+F | Decrease | Reduce requests for radiological tests on lumbar spine and knee radiographs | The number of each radiograph requested per 1000 patients registered with every practice per year for 2 years; the second year was the intervention period. | General practitioner | cRCT |
| Everett 1983 | A+F | Decrease | Decrease the use of laboratory services | The unit of statistical analysis consisted of the number of tests performed | Internal medicine physicians | cRCT |
| Everitt 1990 | AB | Decrease | Decrease inappropriate perioperative prophylaxis antibiotics | Relative use of Cefazolin or cefoxitin in caesarean sections that received <5g of either drug perioperatively. | Physicians in Obstetrics & Gynaecology | ITS |
| Fairbrother 1999 | A+F | Increase | Improve immunization rates | Up to date immunization status | Physicians | cRCT |
| Feldstein 2006 | IM | Increase | Increase BMD measurement and osteoporosis medication. | BMD measurement and osteoporosis medication. | Primary care physicians | RCT |
| Feldstein 2007 | IM | Increase | Improve management of osteoporosis after a fracture either by BMD or treatment | Proportion of patients who received BMD within 6 mos. post-fracture; proportion who had Osteoporosis medication prescribed | Family Physicians | RCT |
| Ferguson 2003 | A+F | Increase | Increase use of process measures in patients undergoing coronary artery bypass graft surgery | Use of process of care measures: Beta-blockage and internal mammary artery grafting | Health Professionals in hospital | cRCT |
| Fine 2003 | AB | Decrease | Reduce duration of Intravenous Antibiotic | Duration of intravenous antibiotic therapy | Physicians | cRCT |
| Foster 2007 | A+F | Increase | Improve management of asthma (recording peak flow in chart) | Number of patients with asthma with peak flow recordings | Primary care physicians who deliver most pneumonia care | CBA |
| Foy 2004 | AB | Increase | Prescribe antibiotic prophylaxis | Odds ratio of receiving prophylactic antibiotics for lower genital tract infection | Clinician in gynaecological unit | cRCT |
| Foy 2004a | A+F | Increase | Screening for lower genital tract infection | Screening for lower genital tract infection | Clinicians | cRCT |
| Franz 2004 | AB | Decrease | Reduce antibiotic therapy | Proportion of infants treated with antibiotics within 7 days after study entry of all infants enrolled for suspected infection | Physicians in neonatology | RCT |
| Fraser 1997 | AB | Decrease | Prescribe appropriate antibiotic, dosing regimen or timing | Antibiotic charges | Physicians in Medical, surgery, intensive care, haematology and oncology | RCT |
| Fridkin 2002 | AB | Decrease | Reduce vancomycin use | Vancomycin use; presence of vancomycin-resistant enterococci | Physicians in ICU | CBA |
| Frijling 2002 | A+F | Increase | Improve decision making in diabetes care | Compliance rates with evidence-based recommendations pertaining to discussion of body weight control, discussion of problems with medication, blood pressure measurement, foot examination, eye examination, initiating anti-diabetic medication or increasing the dosage in cases of uncontrolled blood glucose, and scheduling a follow-up appointment | Family physicians/ GP | cRCT |
| Frijling 2003 | A+F | Increase | Increase frequency of 12 behaviours | Clinical decision making of GP's in cardiovascular care (12 Behaviours assessment of risk factors for Newly diagnosed hypertension) | Family physicians/ GP | cRCT |
| Gama 1992 | A+F | Decrease | Decrease laboratory test usage | Clinical chemistry test requests, revenue expenditure | Consultant physicians (3 intervention; 2 control) | cRCT |
| Gardner 2005 | IM | Increase | Increase the follow-up for a hip fracture by BMD ordered and anti-resorptive Therapy. | The ratio of the number of patients in each group to the number of patients in whom their primary physician had addressed the osteoporosis, as reported by the patient. | Primary care physicians | RCT |
| Gehlbach 1984 | A+F | Increase | Increase generic drug prescribing | Increase generic drug prescribing | Family physicians/ GP | cRCT |
| Gerding 1985 | AB | Decrease | Change from one antibiotic to another | Aminoglycoside use and resistance to gentamicin | Physicians in 14 hospitals | ITS, UBA |
| Goff 2003 | A+F | Increase | Increase use of coronary heart disease medications | The use of 3-hydroxy- 3methylglutaryl coenzyme A (HMG CoA) reductase inhibitors, beta blockers, and angiotensin-converting enzyme (ACE) inhibitors in patients with CHD | Primary physicians | cRCT |
| Grady 1997 | A+F | Increase | Increase mammography referral | Quarterly and annual mammography referral rates, Quarterly and annual mammography completion rates | Primary care physicians | cRCT |
| Guadagnoli 2000 | A+F | Increase | Improve discussion of surgical treatment options for patients with breast cancer | Proportion of patients who report surgeon didn't discuss surgical options | Surgeons | cRCT |
| Gupta 1989 | AB | Decrease | Reduce Cefazolin usage | Percentage of order that complied with extended intervals | Health professional | ITS |
| Halm 2004 | AB | Increase | Improve the quality, efficiency, and patient understanding of care for community-acquired pneumonia | Percentage of patients treated with appropriate antibiotic | Hospital Physicians | ITS |
| Hayes 2001 | A+F | Increase | Improve care of inpatients with venous thrombosis | Nuclear medicine—ventilation perfusion lung scans Routine INR reporting with prothrombin times Use of heparin nomogram, standard orders, or protocol | Internists | RCT |
| Hayes 2002 | A+F | Increase | Adhere to congestive heart failure guidelines (guidelines used) four indicators of increase in behaviour | Documentation of left ventricle function, Use of ACE inhibitors, Use of target dose of ACE inhibitors, Use of warfarin. | Internists | cRCT |
| Herbert 2004 | A+F | Increase | Improve prescribing for hypertension | Increased prescriptions of thiazide | Family physicians/ GP | cRCT |
| Hershey 1986 | A+F | Decrease | Reduce prescribing charges | Mean number of prescriptions per patient. | Residents in four firms in a US hospital | cRCT |
| Hershey 1988 | A+F | Decrease | Reduce total outpatient prescribing charges | Outpatient pharmacy charges | Residents in four units in a US hospital | cRCT |
| Hess 1990 | AB | Decrease | Reduce antimicrobial agents | Total expenditure for antimicrobial agents | Hospital staff in US | ITS |
| Hillman 1998 | A+F | Increase | Increase compliance to cancer screening | Mammography, breast exam, colorectal screening, and Pap testing compliance rates were evaluated | Primary care physicians | cRCT |
| Hillman 1999 | A+F | Increase | Comply with paediatric care guidelines (immunization + other) | Increase a number of behaviours | Family physicians/ GP/ paediatricians | cRCT |
| Himmelberg 1991 | AB | Increase | Increase antibiotic usage | Quantity of Antimicrobial Use | Hospital physicians excluding paediatric department | ITS |
| Hollingworth 2006 | IM | Decrease | Reduce unnecessary radiographs by limiting referrals to patients based on guidelines | Primary care requests for lumbar spine radiography from computerised records | Primary care Professionals | ITS |
| Holm 1990 | A+F | Decrease | Decrease prescription rate of hypnotics/sedatives | The prescription rate was recorded before and after the intervention. | General Practitioners | cRCT |
| Huber 1982 | AB | Decrease | Decrease benzodiazepines and cephalosporins | Dosage units dispensed | Physicians in large US hospital | ITS |
| Hulgan 2004 | AB | Increase | Increase the proportion of oral quinolone antibiotic orders | Proportion of oral quinolone antibiotic orders placed for hospitalized patients. | Health care professionals at university hospital in the south-eastern US | ITS |
| Hux 1999 | A+F | Decrease | Improve antibiotic use | Decrease prescription cost. | Family physicians/ GP | cRCT |
| Inaraja 1986 | AB | Decrease | Reduce cephalosporins | Frequency of use of each group of antimicrobial agents | Health professionals in university hospital in Spain | ITS |
| Jackson 2005 | IM | Decrease | Improve management of Acute low back pain | Number of radiographs ordered for LBP; medication recommendations (acetaminophen, NSAIDs, muscle relaxants, narcotics); physiotherapy referrals | Primary care physicians | ITS |
| Kahan 2009 | A+F | Increase | Adhere to a guideline for the treatment of acute uncomplicated cystitis in women | Increase prescribing of nitrofurantoin | Family physicians/ GP/ internists | cRCT |
| Kerry 2000 | A+F | Decrease | decreasing X-ray referral in GP's | Requests for spinal examinations | General Practitioners | cRCT |
| Kerry 2000a | IM | Decrease | Reduce the number of GP radiological requests | Requests for spinal examinations | General Practitioners | cRCT |
| Khan 2003 | AB | Decrease | Reduce use of cephalosporins | Incidence of *C.Diff.* | Anyone who prescribes cephalosporins in hospital | ITS |
| Kiefe 2001 | A+F | Increase | Improve diabetes care | Number of behaviours | Family physicians / GP / internists | cRCT |
| Kim 1999 | A+F | Increase | Improve quality of care by GP's for those 65-75 years old | Provide advice about Influenza vaccine Pneumococcal vaccine; Tetanus vaccine Mammography Breast examination Exercise counselling Smoking cessation | Family physicians/ GP/ internists | cRCT |
| Kinsinger 1998 | A+F | Increase | Improve breast cancer screening rates | Mammography were recorded in two ways mention of the test in the visit note and actual report of the test in the medical record | Physicians and staff of 62 randomly selected family medicine and general internal medicine practices | cRCT |
| Kogan 2003 | A+F | Increase | Improve compliance with preventive health and disease management recommendations | Screening (10) Immunizations (3) Counselling (5) Total preventive health (18) Diabetes management (12) Hypertension management (5) Coronary artery disease management (5) Asthma management (5) | Internists | cRCT |
| Kritchevsky 2008 | A+F | Increase | Prescribe appropriate pre-surgical antimicrobial prophylaxis | The change in the hospital’s proportion of patients receiving appropriately timed prophylaxis, defined as the proportion of patients who received at least 1 prophylaxis dose administered within 60 minutes before incision | Surgeons | RCT |
| Kumana 2001 | AB | Decrease | Reduce unnecessary use of vancomycin and teicoplanin based on CDC Guidelines | Glycopeptides usage data for our hospital and others in Hong Kong were retrieved and analysed as well as samples of records of our inpatients with staphylococcal septicaemia (pre and during ICF). | Health Professionals in hospital in Hong Kong | ITS |
| Lafata 2007 | IM | Increase | Improve osteoporosis screening with BMD test | Screening rates | Primary care physicians in 15 clinics | cRCT |
| Lakshminarayan 2010 | A+F | Increase | Improve stroke care for 10 performance measures | Acute care bundle (3 PERFORMANCE MEASURES); Inpatient care bundle (4 PERFORMANCE MEASURES) Discharge care bundle (3 PERFORMANCE MEASURES); | Family physicians/ GP/ internists | cRCT |
| Landman 1999 | AB | Decrease | Replace one antibiotic for another | Presence of bacteria infection | Hospital Physicians | ITS |
| Lautenbach 2003 | AB | Decrease | Reduce vancomycin, and third generation cephalosporins | Use of vancomycin | Health professional at hospital in US hospital | ITS |
| Lee 1995 | AB | Decrease | Change from one antibiotic to another | Antimicrobial cost savings (1993-1994) and number of recommendations made and accepted JAN-DEC 1994 | Physicians in hospital | ITS |
| Leverstein-van Hall 2001 | AB | Decrease | Reduce antibiotic prescribing for Gram negative infection | Prevalence of gentamicin resistant Enterobacteriaceae; use of antibiotics in DDD/100 bed | Physicians in neurology and neurosurgery divisions | ITS |
| Lobach 1996 | A+F | Increase | Adhere to diabetes guidelines Measure of HBA1c, Flu vaccine, cholesterol | Hba1c or cholesterol influenza | Family physicians/ GP | cRCT |
| Lomas 1991 | A+F | Increase | Improve management of woman with previous caesarean | Increase trial of labour and vaginal birth rates decrease caesarean | Obstetricians | cRCT |
| Madaras-Kelly 2006 | AB | Decrease | Decrease the use of fluoroquinolone antibiotics | Decrease in infection (secondary fluoroquinolone usage) | Physician in a Veterans Affairs (USA) hospital with an extended care facility | ITS |
| Mainous 2000 | A+F | Decrease | Reduce antibiotic prescription | Reduce antibiotic overuse | Primary care physicians | cRCT |
| Majumdar 2004 | IM | Increase | Increase detection / improve rates of BMD testing | The primary end point was the prescription of osteoporosis treatment 6 months after fracture. Secondary end points included rates of testing for bone mineral density | Emergency department physicians in two hospitals in Canada | CCT |
| Majumdar 2007 | IM | Increase | Improve Osteoporosis Treatment After Hip Fracture | Proportion of patients who had BMD testing within 6 mos.; bisphosphonate therapy. | Health professional in a hospital | RCT |
| Majumdar 2008 | IM | Increase | Improve the quality of osteoporosis care. | Occurrence of a bone mineral density test; (secondary measure increase BMD testing) | Primary care physicians | RCT |
| Martin 1980 | A+F | Decrease | Reduce ordering of lab and radiology tests | Number of laboratory and radiology tests | Medical residence in first year post-graduate | cRCT |
| Matowe 2002 | IM | Decrease | Improve referral for radiography | Number of X-ray referrals | Primary care physicians | ITS |
| May 2000 | AB | Decrease | Decrease use of cephalosporins | Use of antibiotics | Health Professionals in a burn and Trauma ICU who prescribe antibiotics | ITS |
| Mayer 1998 | A+F | Increase | Provide skin cancer prevention counselling | Rate of cancer preventative counselling | Pharmacists | cRCT |
| McCartney 1997 | A+F | Increase | Improve prophylactic aspirin prescription | Increase aspirin in patients with heart disease | Family physicians/ GP | RCT |
| McClellan 2003 | A+F | Increase | Monitor glycosylated haemoglobin in Diabetes Mellitus | The measures of the effect of our intervention included changes in frequency of measurement of HbA1C, quantitative urine protein, and dilated eye examinations. | Family physicians/ GP/ internists/obstetricians | cRCT |
| McConnell 1982 | A+F | Decrease | Prescribe Tetracycline | Tetracycline for URTI | Primary care physicians | cRCT |
| McElnay 1995 | AB | Decrease | Reduce antibiotic usage | Expenditure on antibiotics | Physicians from various specialities within hospital | ITS |
| McGowan 1976 | AB | Decrease | Decrease use of restricted antimicrobial drugs | Proportion of Antibiotic expenditures | Physicians in hospital | ITS |
| McLaughlin 2005 | AB | Decrease | Reduce inappropriate intravenous (IV) antibiotic therapy | IV antibiotic duration, appropriateness of IV route, switching | On-call junior medical staff | ITS |
| McNulty 1997 | AB | Decrease | Decrease use of restricted antibiotic drugs | Cefuroxime prescribing | Physicians in elderly care unit | ITS |
| Mercer 1999 | AB | Decrease | Reduce antibiotic use | Antibiotic use and cost data for the 12 -month periods | Health professionals responsible for Antibiotic prescribing | ITS |
| Meyer 1993 | AB | Decrease | Decrease use of Ceftazidime | Use of ceftazidime, imipenem and ceftriaxone. | Physicians at a hospital in USA | ITS |
| Micek 2004 | AB | Decrease | Decrease antibiotics | Duration of antibiotic treatment. | Health professionals responsible for Antibiotic prescribing | RCT |
| Millard 2008 | A+F | Increase | Improve documentation of dementia | Increase dementia diagnosis | Family physicians/ GP | cRCT |
| Mitchell 2005 | A+F | Increase | Manage of hypertension in GP | Increase number of patients with identified, treated, and controlled hypertension | Family physicians/ GP | cRCT |
| Moher 2001 | A+F | Increase | Increase Secondary prevention of coronary artery disease | Increase assessment of Cholesterol levels, reporting of smoking status, in blood pressure, prescribing of hypotensive, lipid lowering, or antiplatelet agents, | Family physicians/ GP/ nurses | cRCT |
| Mold 2008 | A+F | Increase | Increase preventative services in primary care | Rates in a number of preventative services (DTaP#4, measles/mumps/rubella, HepB#3, Pneumovax, mammography, and colorectal cancer screening). | Family physicians | cRCT |
| Naughton 2001 | AB | Increase | Increase use of parenteral antibiotic for treatment of nursing home pneumonia | Antibiotic use at diagnosis | Health professionals in 10 skilled nursing facilities in the US | RCT |
| Naughton 2007 | A+F | Increase | Prescribe preventative cardiovascular disease therapy | Increase prescription for patients with CVD | Family physicians/ GP | RCT |
| Naughton 2009 | A+F | Decrease | Reduce: the overall rate of antibiotic and proportion of second-line antibiotic prescribing. | Rate of prescription and proportion of second-line antibiotic prescription | Family physicians/ GP | cRCT |
| Nilsson 2001 | A+F | Both (multiple behaviours) | Improve prescribing behaviour | Prescribing of beta-blocking agents and diuretics; increase prescribing in general and per prescription and the prescribing of H2-receptor antagonists at the expense of PPIs | Family physicians/ GP | RCT |
| O'Connor 2009 | A+F | Increase | Improve safety and quality of diabetes care | A1C and LDL cholesterol testing rates | Family physicians/ GP/ internists | cRCT |
| Oakeshott 1994 | IM | Decrease | Reduce the use of clinically unhelpful X-ray examinations. | Number of X-rays requested | General Practitioners | cRCT |
| Oosterheert 2005 | AB | Decrease | Reduce unnecessary antibiotic use | Duration of treatment (days) | Health professionals in a university hospital in the Netherlands | RCT |
| Palmer 1985 | A+F | Increase | Improve care for 4 medical, 4 paediatric tasks | Follow-up of a new finding of a hematocrit of 34 or less in women and 38 or less in men; Performance of annual Pap smears and breast examinations; Follow-up of serum glucose of 200 mg/dl or more in adults; Monitoring of adult patients on digoxin; Follow-up of a new finding of a positive urine culture in children 6 months to 16 years of age; Compliance with selected well childcare standards of the American Academy of Paediatrics; Assessment of the risk of dehydration; follow-up of children 8 years of age and younger to detect resolution of middle ear effusion in an episode of otitis media | Internist / paediatricians | cRCT |
| Patel 1989 | AB | Decrease | Reduce augmentin antibiotics | Expenditure of oral co-amoxiclav. | Hospital Physicians | ITS |
| Paul 2006 | AB | Decrease | Reduce inappropriate antibiotic use | Appropriate antibiotic treatment, was assessed among patients with microbiologically documented infections (MDI).. | Health professionals in hospitals in Israel, Germany and Italy | cRCT |
| Pear 1994 | AB | Decrease | Decrease use of clindamycin | Cases of *C. dif*. associated diarrhoea; Prevalence of clindamycin-resistant *C. dif*. | Physicians in hospital | ITS |
| Perez 2003 | AB | Decrease | Reduce incorrect prescribing of antibiotics | Rate of incorrect prescriptions | Physicians, surgeons, paediatricians, obstetricians-gynaecologists and intensivists | ITS |
| Pimlott 2003 | A+F | Decrease | Prescribe of benzodiazepines appropriately to elderly patients | Reduction in long acting benzodiazepine prescriptions for elderly patients | Family physicians/ GP | cRCT |
| Prihar 2008 | IM | Increase | Increase BMD screening in women over 65 | BMD screening rates | Residents in academic internal medicine clinics | cRCT |
| Quinley 2004 | A+F | Increase | Improve vaccination rates | The change in cumulative pneumococcal vaccination rates | Primary care physicians | cRCT |
| Richards 2003 | AB | Decrease | Reduce cefotaxime or ceftriaxone | Change in rate of CEFX | All physicians in the hospital | ITS |
| Richardson 2000 | AB | Decrease | Reduce inappropriate use of vancomycin according to hospital guidelines | Percentage of episodes of vancomycin use deemed inappropriate. | All physicians in the hospital | ITS |
| Rossignol 2000 | IM | Decrease | Improve the treatment of sub-acute low-back pain patients. Decrease use of X-rays | Proportion of patients who received Imaging (X-ray, Ct, MRI, or myelogram) within 6 months | Primary care Professionals | RCT |
| Rozental 2008 | IM | Increase | Improve low evaluation rates (BMD tests) for osteoporosis | Rate of BMD testing | Orthopaedic surgeon and primary care physicians | RCT |
| Rust 1999 | A+F | Increase | Increase immunization | The immunization level of 2-year-old children in the resident clinic was the main outcome of interest | Postgraduate level 2 and postgraduate level 3 paediatric residents | cRCT |
| Saizy-Callaert 2003 | AB | Decrease | Reduce prescribing of most expense antibiotic | Anti-Infective Expenditure (AIE) per hospital patient. | All physicians in the hospital | ITS |
| Salama 1996 | AB | Decrease | Reduce inappropriate antimicrobial use to reduce cost | Antimicrobial cost | Health professionals in hospital | ITS |
| Sandbaek 1999 | A+F | Increase | Increase consultation for AIDS prevention | Frequencies of consultations | Family physicians/ GP | cRCT |
| Schectman 1995 | A+F | Increase | Improve H2-blocker prescribing patterns | Physicians’ cimetidine-prescribing rates | Family physicians/ GP/ internists | RCT |
| Schectman 2003 | A+F | Decrease | Utilize radiologic services for acute low back pain | Proportion of lumbar spine X-rays, CT or MRI consistent with guidelines within 12 mos. | Primary care physicians | RCT |
| Schectman 2003a | IM | Decrease | Utilize specialty services for acute low back pain | Proportion of subspecialty referrals, physiotherapy referrals. | Primary care physicians | RCT |
| Scholes 2006 | A+F | Increase | Increase chlamydia screening | Chlamydia screening in young women | Primary care physicians | cRCT |
| Shojania 1998 | AB | Decrease | Reduce intravenous vancomycin use. | Number of vancomycin orders and duration of vancomycin therapy | Physicians in a tertiary care teaching hospital. | RCT |
| Singh 2000 | AB | Decrease | Minimize unnecessary / overtreatment antibiotic use | Duration of antibiotic treatment. | Physicians in ICU | RCT |
| Sirinavin 1998 | AB | Decrease | Reduce inappropriate / overuse prescribing of antibiotics | Total restricted drugs cost | All physician in hospital | ITS |
| Siriwardena 2002 | A+F | Increase | Increase influenza and pneumococcal vaccinations | Vaccination rates by practices for patients aged 65 years and over, and patients with CHD, diabetes, and splenectomy, six months after the educational outreach visit. | Primary care physicians | cRCT |
| Skaer 1993 | AB | Decrease | Reduce inappropriate / overuse prescribing of antibiotics | Rates of antibiotic use | Health professionals in US hospital | ITS |
| Solomon 2001 | AB | Decrease | Reduce inappropriate /misuse prescribing of antibiotics | Number of days of unnecessary ceftazidime or levofloxacin. | Internal medicine | RCT |
| Solomon 2004 | IM / A+F | Increase | Improve management of glucocorticoid-induced osteoporosis (GIOP) | Bone Density Screening and prescribing Osteoporosis drugs for Glucocorticoid induced osteoporosis | Primary care physicians | RCT |
| Solomon 2007 | IM | Increase | Increase screening (BMD tests) for osteoporosis | The primary outcome studied was a composite of either undergoing a BMD test or initiating a medication used for osteoporosis. | Primary care physicians | cRCT |
| Solomon 2007a | IM | Increase | Improve suboptimal management of osteoporosis as measure by BMD tests and treatment | Either undergoing a bone mineral density (BMD) testing or filling a prescription for a bone-active medication during the 10 months of follow-up. | Primary Care Physicians | cRCT |
| Sondergaard 2002 | A+F | Increase | Improve prescribing patters for asthma drugs | Prescribing inhaled corticosteroid with bets 2 agonists for asthma patients | Family physicians/ GP | RCT |
| Sondergaard 2003 | A+F | Decrease | Improve antibiotic prescription | Antibiotic prescribing for respiratory tract infections | Family physicians/ GP | cRCT |
| Sondergaard 2006 | A+F | Increase | Improve secondary prevention of ischemic heart disease | The proportion of patients being treated with lipid-lowering drugs and acetylsalicylic acid and the frequency of cholesterol measuring and counselling on exercise, smoking cessation and diet | Primary Care Physicians | cRCT |
| Stock 1998 | IM | Increase | Increase use of bone densitometry | Number of BMD tests ordered | Primary Care Physicians | cRCT |
| Suwangool 1991 | AB | Decrease | Reduce antibiotic prescribing | Cost of antibiotics | Physicians in Dept. Medicine | ITS |
| Thomas 2006 | A+F | Decrease | Decrease laboratory tests (9 tests targeted) | Number of each target test requested per practice - 12 months before and after intervention | Family Practitioners | cRCT |
| Thomas 2007 | A+F | Increase | Improve diabetes care processes | Increase haemoglobin A1c (within 6mos) or cholesterol testing (within 1 year). | Internists | cRCT |
| Tierney 1986 | A+F | Increase | Orde preventative care measures (13 protocols developed) | Fecal Blood Testing; Pneumococcal Vaccination; Antacids; TB Skin Testing; Beta blocker; Nitrates; Antidepressant; Calcium Supplements; Cervical Cytology; Mammography; Metronidazole; Digitalis; Salicylates | Internists | RCT |
| Toltzis 1998 | AB | Decrease | Reduce ceftazidime prescribing | Ceftazidime doses | All physicians in a mixed medical and surgical paediatric ICU | ITS |
| Tu 2009 | A+F | Increase | Improve the quality of cardiac care | 12 indicators for acute myocardial infarction and 6 for Congestive Heart Failure | Internists | cRCT |
| Van Kasteren 2005 | AB | Decrease | Reduce the quantity of surgical prophylaxis | Process outcome parameters were antibiotic choice, duration, timing, antibiotic volume and costs. | Health professionals within 13 hospitals in Netherlands | ITS |
| Veninga 1999 | A+F | Increase | Improve treatment of asthma | Prescribing inhaled corticosteroids; continuous bronchodilator monotherapy; inadequate level of inhaled corticosteroids; oral corticosteroids for exacerbations | Family physicians/ GP | cRCT |
| Verstappen 2003 | IM | Decrease | Decrease unnecessary test ordering according to evidence-based guidelines | Total numbered of tests ordered per clinical condition | Primary care physicians | cRCT |
| Verstappen 2004 | A+F | Decrease | Decrease unnecessary test ordering | Total numbered of tests ordered | Primary care physicians | cRCT |
| Verstappen 2004a | A+F | Decrease | Decrease test ordering | The mean number of tests per physician per 6 months was the dependent variable. | Primary care physicians | cRCT |
| Wadland 2007 | A+F | Increase | Improve referral to a tobacco cessation quit line | Number of referrals to a tobacco cessation quit line | 171 family medicine, 88 internal medicine, 49 obstetrics-gynaecology from 87 primary care practices | cRCT |
| Weinberg 2001 | AB | Increase | Increase antibiotic prescribing so that all women receive prophylaxis | Percentage of women receive prophylaxis; percentage of women who receive prophylaxis within one hour. | Health professional in a hospital | ITS |
| Wilson 1991 | AB | Decrease | Change one drug for another | Use of amoxicillin and pivampicillin | Health professional in a hospital | ITS |
| Winickoff 1984 | A+F | Increase | Improve colorectal cancer screening | Rate of colorectal screening | Internal Medicine physicians | cRCT |
| Winkens 1995 | A+F | Decrease | Reduce numbers of test requests | Proportion of requests | Family doctors | cRCT |
| Winkens 1995 | IM | Decrease | Reduce numbers of tests | Proportion of tests ordered | Primary care physicians | cRCT |
| Wones 1987 | A+F | Decrease | Reduce laboratory test utilization | Number of tests per patient-day | Internists | RCT |
| Woodward 1987 | AB | Decrease | Reduce antibiotic prescribing | Total antibiotic costs | Clinical health professionals in hospital | ITS |
| Wyatt 1998 | AB | Increase | Increase in use of antibiotic prophylaxis for caesarean section | Percentage of caesarean sections that received antibiotic prophylaxis. | Lead obstetrician and senior midwife manager in 25 hospitals | cRCT |
| Young 1985 | AB | Decrease | Decrease use of gentamicin | Aminoglycoside usage | Health professionals responsible for Antibiotic prescribing | ITS |
| Zanetti 2003 | AB | Increase | Increase in use of antibiotic for cardiac surgery patients | Percentage of patients who received additional intra-operative antibiotics | Clinicians involved in cardiac surgery | RCT |
| Zwar 1999 | A+F | Decrease | Decrease antibiotics for upper respiratory infections | Number of prescriptions per 100 URTI problems | General Practice trainees in New South wales | RCT |
