# Supplementary material for: Changing behaviour, ‘more or less’: do implementation and de-implementation interventions include different behaviour change techniques?
Source: Implement Sci. 2021 Feb 25;16:20. doi: 10.1186/s13012-021-01089-0 (PMC7905859; doi:10.1186/s13012-021-01089-0)
Supplement: Supplementary file 3 — Additional file 3. Supplemental File 3 Sample of BCT coding of intervention description [file 13012_2021_1089_MOESM3_ESM.docx]

Supplemental File #3: Sample of BCT Coding of Intervention Description
