# Supplementary material for: Changing behaviour, ‘more or less’: do implementation and de-implementation interventions include different behaviour change techniques?
Source: Implement Sci. 2021 Feb 25;16:20. doi: 10.1186/s13012-021-01089-0 (PMC7905859; doi:10.1186/s13012-021-01089-0)
Supplement: Supplementary file 4 — Additional file 4. Supplemental File 4 BCTs identified in intervention descriptions [file 13012_2021_1089_MOESM4_ESM.docx]

Supplemental File #4: BCTs identified in intervention descriptions; Implementation Interventions (n = 81)

|  | Goal Setting (behaviour) | Problem Solving | Goal Setting (outcome) | Action Planning | Review of behaviour goals | Discrepancy between current behaviour and goal | Behavioural Contract | Commitment | Monitoring of behaviour by others without feedback | Feedback on behaviour | Self-monitoring of behaviour | Feedback on outcomes of behaviour | Social Support (unspecified) | Social Support (Practical) | Instruction on how to perform the behaviour | Information about health consequences | Information about social and environmental consequences | Demonstration of the behaviour | Social Comparison | Prompts / cues | Behavioural practice / rehearsal | Behaviour Substitution | Credible Source | Pros and Cons | Comparative imagining of future outcomes | Material Incentive (behaviour) | Material reward (behaviour) | Non-specific Incentive | Restructuring Physical Environment | Adding Object to the environment |
| --- | --- | --- | --- | --- | --- | --- | --- | --- | --- | --- | --- | --- | --- | --- | --- | --- | --- | --- | --- | --- | --- | --- | --- | --- | --- | --- | --- | --- | --- | --- |
| Anderson 1994 | 1 |  |  |  |  |  |  |  |  | 1 |  |  |  | 1 | 1 |  |  |  |  | 1 |  |  |  |  |  |  |  |  |  |  |
| Baker 2003a |  |  |  |  |  |  |  |  |  | 1 |  |  |  |  | 1 |  |  |  | 1 |  |  |  |  |  |  |  |  |  |  |  |
| Balas 1998 |  |  |  |  |  |  |  |  |  | 1 |  |  |  |  |  | 1 | 1 |  |  |  |  |  |  |  |  |  |  |  |  |  |
| Barlow 2007 |  |  |  |  |  |  |  |  |  |  |  |  |  |  | 1 |  |  |  |  |  |  |  |  | 1 |  |  |  |  |  |  |
| Beck 2005 |  |  |  |  |  |  |  |  |  | 1 |  |  |  |  |  |  |  |  |  |  |  |  |  |  |  |  |  |  |  |  |
| Bentz 2007 |  |  |  |  |  |  |  |  |  | 1 |  |  |  |  |  |  |  |  | 1 |  |  |  |  |  |  |  |  |  |  |  |
| Bonevski 1999 | 1 |  |  |  | 1 |  |  |  |  | 1 |  |  |  |  | 1 | 1 |  |  |  |  |  |  |  |  |  |  |  |  |  |  |
| Boyd 2002 |  |  |  |  |  |  |  |  |  |  |  |  |  |  | 1 | 1 |  |  |  |  |  |  |  |  |  |  |  |  |  |  |
| Brady 1988 |  |  |  |  |  |  |  |  |  |  | 1 |  |  |  | 1 |  |  |  |  |  |  |  |  |  |  |  |  |  |  |  |
| Brown 1994 |  |  |  |  |  |  |  |  |  | 1 |  |  | 1 | 1 | 1 |  |  |  | 1 |  |  |  | 1 |  |  |  |  |  |  |  |
| Buffington 1991 |  |  |  |  |  |  |  |  |  |  | 1 |  |  |  |  |  |  |  |  | 1 |  |  |  |  |  |  |  |  |  | 1 |
| Chu 2003 |  |  |  |  |  |  |  |  |  | 1 |  |  |  |  |  |  |  |  | 1 |  |  |  |  |  |  |  |  |  |  |  |
| Cline 2007 |  |  |  |  |  |  |  |  |  | 1 |  |  |  |  |  |  |  |  |  |  |  |  |  |  |  |  |  |  |  |  |
| Curtis 2005 |  |  |  |  |  |  |  |  |  | 1 |  |  |  |  | 1 |  |  |  | 1 |  |  |  |  |  |  |  |  |  |  |  |
| Curtis 2007 |  |  |  |  |  |  |  |  |  | 1 |  |  |  |  | 1 |  |  |  | 1 |  |  |  |  |  |  |  |  |  |  |  |
| Curtis 2007a |  |  |  |  |  |  |  |  |  |  |  |  |  |  |  |  |  |  |  |  |  |  |  |  |  |  |  |  |  |  |
| Fairbrother 1999 |  |  |  |  |  |  |  |  |  | 1 |  |  |  |  |  |  |  |  | 1 |  |  |  |  |  |  |  | 1 |  |  |  |
| Feldstein 2006 |  |  |  |  |  |  |  |  |  |  |  |  | 1 |  | 1 | 1 | 1 |  |  | 1 |  |  |  |  |  |  |  |  | 1 |  |
| Feldstein 2007 |  |  |  |  |  |  |  |  |  |  |  |  |  |  | 1 |  |  |  |  |  |  |  |  |  |  | 1 |  |  |  | 1 |
| Foster 2007 |  | 1 |  |  |  |  |  |  |  | 1 |  |  |  |  | 1 |  |  |  |  |  |  |  | 1 |  |  |  |  |  |  |  |
| Foy 2004 |  |  |  |  |  |  |  |  |  |  |  |  |  |  |  |  |  |  |  |  |  |  |  |  |  |  |  |  |  |  |
| Foy 2004a | 1 |  |  |  |  |  |  |  |  | 1 |  |  |  |  | 1 |  |  |  |  |  |  |  | 1 |  |  |  |  |  |  |  |
| Frijling 2002 | 1 |  | 1 |  | 1 | 1 |  |  |  | 1 |  |  | 1 |  |  |  |  |  |  |  |  |  |  |  |  |  |  |  |  |  |
| Frijling 2003 | 1 |  |  |  | 1 | 1 |  |  |  | 1 |  |  | 1 | 1 |  |  |  |  |  |  |  |  |  |  |  |  |  |  |  |  |
| Gardner 2005 |  |  |  |  |  |  |  |  |  |  |  |  |  |  |  |  |  |  |  | 1 |  |  |  |  |  |  |  |  |  |  |
| Gehlbach 1984 |  |  |  |  |  |  |  |  |  | 1 |  | 1 |  |  |  |  | 1 |  |  |  |  | 1 |  |  |  |  |  |  |  |  |
| Goff 2003 |  |  |  |  |  |  |  |  |  | 1 |  |  |  |  | 1 |  |  |  | 1 | 1 |  |  |  |  |  |  |  |  |  |  |
| Grady 1997 |  |  |  |  |  |  |  |  |  | 1 |  |  |  |  | 1 | 1 |  |  | 1 | 1 |  |  |  |  |  |  | 1 |  |  |  |
| Guadagnoli 2000 |  |  |  |  |  |  |  |  |  |  |  | 1 |  |  | 1 |  |  |  |  |  |  |  | 1 |  |  |  |  |  |  |  |
| Halm 2004 | 1 |  |  |  |  | 1 |  |  |  |  |  |  |  |  | 1 |  |  |  |  |  |  |  |  | 1 |  |  |  |  |  |  |
| Hayes 2001 |  |  |  |  |  |  | 1 |  |  | 1 |  |  | 1 |  | 1 |  |  |  | 1 |  |  |  | 1 | 1 |  | 1 |  |  |  |  |
| Hayes 2002 |  |  |  |  |  |  | 1 |  |  | 1 |  |  | 1 |  | 1 |  |  |  | 1 |  |  |  | 1 | 1 |  |  |  | 1 |  |  |
| Herbert 2004 |  |  |  |  | 1 |  |  |  |  | 1 |  |  |  | 1 | 1 |  |  |  |  |  |  |  |  |  |  |  |  |  |  |  |
| Hillman 1998 |  |  |  |  |  |  |  |  |  | 1 |  |  |  |  |  |  |  |  | 1 |  |  |  |  |  |  |  | 1 |  |  |  |
| Hillman 1999 |  |  |  |  |  |  |  |  |  | 1 |  |  |  |  |  |  |  |  | 1 |  |  |  |  |  |  |  | 1 |  |  |  |
| Hulgan 2004 |  |  |  |  |  |  |  |  |  |  |  |  |  |  |  |  |  |  |  |  |  | 1 |  |  |  |  |  |  | 1 |  |
| Kahan 2009 |  |  |  |  |  |  |  |  |  | 1 |  | 1 |  |  | 1 |  |  |  |  |  |  | 1 |  |  |  |  |  |  |  |  |
| Kiefe 2001 | 1 |  | 1 |  |  |  | 1 |  |  | 1 |  |  |  |  |  |  |  |  | 1 |  |  |  |  |  |  |  |  |  |  |  |
| Kim 1999 |  |  |  |  |  |  |  |  |  | 1 |  |  |  |  | 1 |  |  |  | 1 |  |  |  |  |  |  |  |  |  |  |  |
| Kinsinger 1998 | 1 |  |  | 1 |  |  |  |  |  | 1 |  |  | 1 |  |  |  |  |  |  |  |  |  |  |  |  |  |  |  |  |  |
| Kogan 2003 |  |  |  |  |  |  |  |  |  | 1 |  |  |  |  |  |  |  |  | 1 |  |  |  | 1 |  |  |  |  |  |  |  |
| Kritchevsky 2008 | 1 | 1 |  |  | 1 |  |  |  |  | 1 |  |  | 1 | 1 | 1 |  |  |  |  |  |  |  | 1 |  |  |  |  |  |  |  |
| Lafata 2007 |  |  |  |  |  |  |  |  |  |  |  |  |  |  | 1 |  |  |  |  | 1 |  |  |  |  |  |  |  |  |  |  |
| Lakshminarayan 2010 |  | 1 |  |  |  |  |  |  |  | 1 |  |  |  |  |  |  |  |  |  |  |  |  |  |  |  |  |  |  |  |  |
| Lobach 1996 |  |  |  |  | 1 |  |  |  |  | 1 |  |  |  |  |  |  |  |  |  |  |  |  |  |  |  |  |  |  | 1 |  |
| Lomas 1991 | 1 |  |  |  |  | 1 |  |  | 1 | 1 | 1 |  |  |  |  |  |  |  |  |  |  |  |  |  |  |  |  |  |  |  |
| Majumdar 2004 |  |  |  |  |  |  |  |  |  |  |  |  |  |  |  | 1 |  |  |  |  |  |  | 1 | 1 | 1 |  |  |  |  |  |
| Majumdar 2008 |  |  |  |  |  |  |  |  |  |  |  |  |  |  | 1 | 1 |  |  |  | 1 |  |  | 1 |  |  |  |  |  |  |  |
| Mayer 1998 |  |  |  |  |  |  |  |  |  | 1 |  |  | 1 |  | 1 |  |  |  | 1 |  |  |  |  |  |  |  |  | 1 |  | 1 |
| McCartney 1997 |  |  |  |  |  |  |  |  |  | 1 |  |  | 1 |  | 1 |  |  |  |  |  |  |  |  |  |  |  |  |  |  |  |
| McClellan 2003 |  |  |  |  |  |  |  |  |  | 1 |  |  |  |  | 1 |  |  |  | 1 | 1 |  |  | 1 |  |  |  |  |  |  |  |
| Millard 2008 |  |  |  |  |  |  |  |  |  | 1 |  |  |  |  | 1 |  |  |  |  |  |  |  |  |  |  |  |  |  |  |  |
| Mitchell 2005 |  |  |  |  |  |  |  |  |  | 1 |  |  |  |  |  | 1 |  |  | 1 |  |  |  |  |  |  |  |  |  |  |  |
| Moher 2001 | 1 |  |  |  |  |  |  |  |  | 1 |  |  |  | 1 | 1 |  |  |  |  |  |  |  | 1 |  |  |  |  |  |  |  |
| Naughton 2001 |  | 1 |  |  |  |  |  | 1 |  |  |  |  | 1 |  | 1 |  |  |  |  | 1 |  |  | 1 |  |  |  |  |  |  | 1 |
| Naughton 2007 |  |  |  |  |  |  |  |  |  | 1 |  |  | 1 |  | 1 |  |  |  |  |  |  |  | 1 |  |  |  |  |  |  |  |
| O'Connor 2009 |  |  |  |  |  |  |  |  |  | 1 |  | 1 |  |  | 1 |  |  |  | 1 |  |  |  |  |  |  |  |  |  |  |  |
| Palmer 1985 | 1 |  |  |  | 1 | 1 |  |  |  | 1 |  |  |  |  | 1 |  |  |  |  |  |  |  | 1 |  |  |  |  |  |  |  |
| Prihar 2008 |  |  |  |  |  |  |  |  |  |  |  |  |  |  |  |  |  |  |  | 1 |  |  |  |  |  |  |  |  |  |  |
| Quinley 2004 |  |  |  |  |  |  |  |  |  | 1 |  |  |  |  | 1 |  |  |  | 1 | 1 |  |  | 1 |  |  | 1 |  |  |  |  |
| Rozental 2008 |  |  |  |  |  |  |  |  |  |  |  |  |  |  | 1 |  |  |  |  |  |  |  |  |  |  |  |  |  |  |  |
| Rust 1999 |  |  |  |  |  |  |  |  |  | 1 |  |  |  |  | 1 | 1 |  |  |  |  |  |  |  |  |  |  |  |  |  |  |
| Sandbaek 1999 |  |  | 1 |  | 1 |  |  |  |  | 1 |  |  |  |  | 1 |  |  |  |  | 1 |  |  |  |  |  |  |  |  |  |  |
| Schectman 1995 |  |  |  |  |  |  |  |  |  | 1 |  | 1 |  |  | 1 | 1 |  |  |  |  |  |  |  |  |  |  |  |  |  |  |
| Scholes 2006 |  |  |  |  |  |  |  |  |  | 1 |  |  |  |  | 1 |  |  |  |  |  |  |  |  | 1 |  |  |  |  |  |  |
| Siriwardena 2002 |  |  |  |  |  |  |  |  |  | 1 |  |  |  |  | 1 |  |  |  | 1 |  |  |  | 1 |  |  |  |  |  |  |  |
| Solomon 2004 |  |  | 1 |  |  |  |  |  |  | 1 |  | 1 |  |  | 1 |  |  |  |  | 1 |  |  |  |  |  |  |  |  |  |  |
| Solomon 2004a |  |  | 1 |  |  |  |  |  |  | 1 |  | 1 |  |  | 1 |  |  |  |  | 1 |  |  |  |  |  |  |  |  |  |  |
| Solomon 2007 |  |  |  |  |  |  |  |  |  |  |  |  |  |  | 1 |  |  |  |  |  |  |  |  |  |  |  |  |  | 1 | 1 |
| Solomon 2007a |  |  |  |  |  |  |  |  |  |  |  |  |  |  | 1 |  |  |  |  | 1 |  |  | 1 |  |  |  |  |  |  |  |
| Sondergaard 2002 |  |  |  |  |  |  |  |  |  | 1 |  | 1 |  |  |  |  |  |  |  |  |  |  |  |  |  |  |  |  |  |  |
| Sondergaard 2006 |  |  |  |  |  |  |  |  |  | 1 |  |  |  |  | 1 |  |  |  | 1 |  |  |  | 1 |  |  |  |  |  |  | 1 |
| Stock 1998 |  |  |  |  |  |  |  |  |  |  |  |  |  |  |  | 1 |  |  |  |  |  |  |  |  |  |  |  |  |  |  |
| Thomas 2007 |  |  |  |  |  |  |  |  |  | 1 |  | 1 |  |  | 1 |  |  |  |  |  |  |  |  |  |  |  |  |  |  |  |
| Tierney 1986 |  |  |  |  |  |  |  |  |  | 1 |  |  |  |  | 1 |  |  |  |  |  |  |  |  | 1 |  |  |  |  |  |  |
| Tu 2009 |  |  |  |  |  |  |  |  |  | 1 |  |  |  |  | 1 |  |  |  | 1 |  |  |  |  |  |  |  |  |  |  |  |
| Veninga 1999 | 1 |  |  |  |  |  |  |  |  | 1 |  |  |  |  | 1 |  |  |  |  |  |  |  |  |  |  |  |  |  |  |  |
| Vernaz 2008 |  |  |  |  |  |  |  |  |  |  |  |  |  |  | 1 |  | 1 |  |  |  |  |  |  |  |  |  |  |  |  |  |
| Wadland 2007 |  |  |  |  |  |  |  |  |  |  |  | 1 |  |  |  |  |  |  | 1 |  |  |  |  |  |  |  |  |  |  |  |
| Weinberg 2001 | 1 | 1 |  | 1 |  |  |  |  |  |  |  |  |  |  |  |  |  |  |  |  |  |  |  |  |  |  |  |  |  |  |
| Whitby 2008 |  |  |  |  |  |  |  |  |  |  |  |  |  |  | 1 |  |  |  |  | 1 |  |  | 1 |  |  |  |  |  |  | 1 |
| Winickoff 1984 | 1 |  |  |  |  | 1 |  |  |  | 1 |  |  |  |  | 1 | 1 |  |  | 1 |  |  |  |  |  |  |  |  |  |  |  |
| Zanetti 2003 |  |  |  |  |  |  |  |  |  |  |  |  |  |  |  |  |  |  |  | 1 |  |  |  |  |  |  |  |  | 1 |  |

De-implementation interventions (n = 93)

| **Studies** | Goal Setting (behaviour) | Problem Solving | Review of behaviour goals | Discrepancy between current behaviour and goal | Monitoring of behaviour by others without feedback | Feedback on behaviour | Self-monitoring of behaviour | Monitoring of outcomes of behaviour without feedback. | Feedback on outcomes of behaviour | Social Support (unspecified) | Social Support (Practical) | Instruction on how to perform the behaviour | Information about health consequences | Information about social and environmental consequences | Demonstration of the behaviour | Social Comparison | Prompts / cues | Behaviour Substitution | Credible Source | Pros and Cons | Material Incentive (behaviour) | Restructuring Physical Environment | Restructuring the Social Environment | Adding Object to the environment |
| --- | --- | --- | --- | --- | --- | --- | --- | --- | --- | --- | --- | --- | --- | --- | --- | --- | --- | --- | --- | --- | --- | --- | --- | --- |
| Abramowitz 1982 |  |  |  |  |  |  |  |  |  |  |  |  |  |  |  |  |  |  |  |  |  |  |  |  |
| Adachi 1997 |  |  |  |  |  |  |  |  |  |  |  |  |  |  |  |  |  |  |  |  |  |  |  |  |
| Ansari 2003 |  |  |  |  |  |  |  |  |  |  |  |  |  |  |  |  |  |  |  |  |  |  |  |  |
| Avorn 1988 |  |  |  |  |  |  |  |  |  |  |  |  |  |  |  |  |  |  |  |  |  |  |  |  |
| Awad 2006 |  |  |  |  |  |  |  |  |  |  |  |  |  |  |  |  |  |  |  |  |  |  |  |  |
| Bailey 1997 |  |  |  |  |  |  |  |  |  |  |  |  |  |  |  |  |  |  |  |  |  |  |  |  |
| Baker 1987 |  |  |  |  |  |  |  |  |  |  |  |  |  |  |  |  |  |  |  |  |  |  |  |  |
| Batty 2001 |  |  |  |  |  |  |  |  |  |  |  |  |  |  |  |  |  |  |  |  |  |  |  |  |
| Belliveau 1996 |  |  |  |  |  |  |  |  |  |  |  |  |  |  |  |  |  |  |  |  |  |  |  |  |
| Berild 2002 |  |  |  |  |  |  |  |  |  |  |  |  |  |  |  |  |  |  |  |  |  |  |  |  |
| Berman 1998 |  |  |  |  |  |  |  |  |  |  |  |  |  |  |  |  |  |  |  |  |  |  |  |  |
| Bradley 1999 |  |  |  |  |  |  |  |  |  |  |  |  |  |  |  |  |  |  |  |  |  |  |  |  |
| Bunz 1990 |  |  |  |  |  |  |  |  |  |  |  |  |  |  |  |  |  |  |  |  |  |  |  |  |
| Calil 2001 |  |  |  |  |  |  |  |  |  |  |  |  |  |  |  |  |  |  |  |  |  |  |  |  |
| Carling 2003 |  |  |  |  |  |  |  |  |  |  |  |  |  |  |  |  |  |  |  |  |  |  |  |  |
| Charbonneau 2006 |  |  |  |  |  |  |  |  |  |  |  |  |  |  |  |  |  |  |  |  |  |  |  |  |
| Chassin 1986 |  |  |  |  |  |  |  |  |  |  |  |  |  |  |  |  |  |  |  |  |  |  |  |  |
| Christ-Crain 2004 |  |  |  |  |  |  |  |  |  |  |  |  |  |  |  |  |  |  |  |  |  |  |  |  |
| Cohen 1982 |  |  |  |  |  |  |  |  |  |  |  |  |  |  |  |  |  |  |  |  |  |  |  |  |
| de Champs 1994 |  |  |  |  |  |  |  |  |  |  |  |  |  |  |  |  |  |  |  |  |  |  |  |  |
| de Man 2001 |  |  |  |  |  |  |  |  |  |  |  |  |  |  |  |  |  |  |  |  |  |  |  |  |
| Dey 2004 |  |  |  |  |  |  |  |  |  |  |  |  |  |  |  |  |  |  |  |  |  |  |  |  |
| Dranitisaris 2001 |  |  |  |  |  |  |  |  |  |  |  |  |  |  |  |  |  |  |  |  |  |  |  |  |
| Eccles 2001 |  |  |  |  |  |  |  |  |  |  |  |  |  |  |  |  |  |  |  |  |  |  |  |  |
| Everett 1983 |  |  |  |  |  |  |  |  |  |  |  |  |  |  |  |  |  |  |  |  |  |  |  |  |
| Everitt 1990 |  |  |  |  |  |  |  |  |  |  |  |  |  |  |  |  |  |  |  |  |  |  |  |  |
| Fine 2003 |  |  |  |  |  |  |  |  |  |  |  |  |  |  |  |  |  |  |  |  |  |  |  |  |
| Franz 2004 |  |  |  |  |  |  |  |  |  |  |  |  |  |  |  |  |  |  |  |  |  |  |  |  |
| Fraser 1997 |  |  |  |  |  |  |  |  |  |  |  |  |  |  |  |  |  |  |  |  |  |  |  |  |
| Fridkin 2002 |  |  |  |  |  |  |  |  |  |  |  |  |  |  |  |  |  |  |  |  |  |  |  |  |
| Gama 1991 |  |  |  |  |  |  |  |  |  |  |  |  |  |  |  |  |  |  |  |  |  |  |  |  |
| Gerding 1985 |  |  |  |  |  |  |  |  |  |  |  |  |  |  |  |  |  |  |  |  |  |  |  |  |
| Gupta 1989 |  |  |  |  |  |  |  |  |  |  |  |  |  |  |  |  |  |  |  |  |  |  |  |  |
| Hershey 1988 |  |  |  |  |  |  |  |  |  |  |  |  |  |  |  |  |  |  |  |  |  |  |  |  |
| Hershey 1986 |  |  |  |  |  |  |  |  |  |  |  |  |  |  |  |  |  |  |  |  |  |  |  |  |
| Hess 1990 |  |  |  |  |  |  |  |  |  |  |  |  |  |  |  |  |  |  |  |  |  |  |  |  |
| Hollingworth 2006 |  |  |  |  |  |  |  |  |  |  |  |  |  |  |  |  |  |  |  |  |  |  |  |  |
| Holm 1990 |  |  |  |  |  |  |  |  |  |  |  |  |  |  |  |  |  |  |  |  |  |  |  |  |
| Huber 1982 |  |  |  |  |  |  |  |  |  |  |  |  |  |  |  |  |  |  |  |  |  |  |  |  |
| Hux 1999 |  |  |  |  |  |  |  |  |  |  |  |  |  |  |  |  |  |  |  |  |  |  |  |  |
| Inaraja 1986 |  |  |  |  |  |  |  |  |  |  |  |  |  |  |  |  |  |  |  |  |  |  |  |  |
| Jackson 2005 |  |  |  |  |  |  |  |  |  |  |  |  |  |  |  |  |  |  |  |  |  |  |  |  |
| Kerry 2000 |  |  |  |  |  |  |  |  |  |  |  |  |  |  |  |  |  |  |  |  |  |  |  |  |
| Kerry 2000a |  |  |  |  |  |  |  |  |  |  |  |  |  |  |  |  |  |  |  |  |  |  |  |  |
| Khan 2003 |  |  |  |  |  |  |  |  |  |  |  |  |  |  |  |  |  |  |  |  |  |  |  |  |
| Kumama 2001 |  |  |  |  |  |  |  |  |  |  |  |  |  |  |  |  |  |  |  |  |  |  |  |  |
| Landman 1999 |  |  |  |  |  |  |  |  |  |  |  |  |  |  |  |  |  |  |  |  |  |  |  |  |
| Lautenbach 2003 |  |  |  |  |  |  |  |  |  |  |  |  |  |  |  |  |  |  |  |  |  |  |  |  |
| Lee 1995 |  |  |  |  |  |  |  |  |  |  |  |  |  |  |  |  |  |  |  |  |  |  |  |  |
| Leverstein-van Hall 2001 |  |  |  |  |  |  |  |  |  |  |  |  |  |  |  |  |  |  |  |  |  |  |  |  |
| Madaras-Kelly 2006 |  |  |  |  |  |  |  |  |  |  |  |  |  |  |  |  |  |  |  |  |  |  |  |  |
| Mainous 2000 |  |  |  |  |  |  |  |  |  |  |  |  |  |  |  |  |  |  |  |  |  |  |  |  |
| Martin 1980 |  |  |  |  |  |  |  |  |  |  |  |  |  |  |  |  |  |  |  |  |  |  |  |  |
| Matowe 2002 |  |  |  |  |  |  |  |  |  |  |  |  |  |  |  |  |  |  |  |  |  |  |  |  |
| May 2000 |  |  |  |  |  |  |  |  |  |  |  |  |  |  |  |  |  |  |  |  |  |  |  |  |
| McConnell 1982 |  |  |  |  |  |  |  |  |  |  |  |  |  |  |  |  |  |  |  |  |  |  |  |  |
| McElnay 1995 |  |  |  |  |  |  |  |  |  |  |  |  |  |  |  |  |  |  |  |  |  |  |  |  |
| McGowan 1976 |  |  |  |  |  |  |  |  |  |  |  |  |  |  |  |  |  |  |  |  |  |  |  |  |
| McLaughlin 2005 |  |  |  |  |  |  |  |  |  |  |  |  |  |  |  |  |  |  |  |  |  |  |  |  |
| McNulty 1997 |  |  |  |  |  |  |  |  |  |  |  |  |  |  |  |  |  |  |  |  |  |  |  |  |
| Mercer 1999 |  |  |  |  |  |  |  |  |  |  |  |  |  |  |  |  |  |  |  |  |  |  |  |  |
| Micek 2004 |  |  |  |  |  |  |  |  |  |  |  |  |  |  |  |  |  |  |  |  |  |  |  |  |
| Naughton 2009 |  |  |  |  |  |  |  |  |  |  |  |  |  |  |  |  |  |  |  |  |  |  |  |  |
| Oakeshott 1994 |  |  |  |  |  |  |  |  |  |  |  |  |  |  |  |  |  |  |  |  |  |  |  |  |
| Oosterheert 2005 |  |  |  |  |  |  |  |  |  |  |  |  |  |  |  |  |  |  |  |  |  |  |  |  |
| Patel 1989 |  |  |  |  |  |  |  |  |  |  |  |  |  |  |  |  |  |  |  |  |  |  |  |  |
| Paul 2006 |  |  |  |  |  |  |  |  |  |  |  |  |  |  |  |  |  |  |  |  |  |  |  |  |
| Pear 1994 |  |  |  |  |  |  |  |  |  |  |  |  |  |  |  |  |  |  |  |  |  |  |  |  |
| Perez 2003 |  |  |  |  |  |  |  |  |  |  |  |  |  |  |  |  |  |  |  |  |  |  |  |  |
| Pimlott 2003 |  |  |  |  |  |  |  |  |  |  |  |  |  |  |  |  |  |  |  |  |  |  |  |  |
| Richards 2003 |  |  |  |  |  |  |  |  |  |  |  |  |  |  |  |  |  |  |  |  |  |  |  |  |
| Richardson 2000 |  |  |  |  |  |  |  |  |  |  |  |  |  |  |  |  |  |  |  |  |  |  |  |  |
| Rossignol 2000 |  |  |  |  |  |  |  |  |  |  |  |  |  |  |  |  |  |  |  |  |  |  |  |  |
| Saizy-Callaert 2003 |  |  |  |  |  |  |  |  |  |  |  |  |  |  |  |  |  |  |  |  |  |  |  |  |
| Salama 1996 |  |  |  |  |  |  |  |  |  |  |  |  |  |  |  |  |  |  |  |  |  |  |  |  |
| Schectman 2003 |  |  |  |  |  |  |  |  |  |  |  |  |  |  |  |  |  |  |  |  |  |  |  |  |
| Schectman 2003a |  |  |  |  |  |  |  |  |  |  |  |  |  |  |  |  |  |  |  |  |  |  |  |  |
| Shojania 1998 |  |  |  |  |  |  |  |  |  |  |  |  |  |  |  |  |  |  |  |  |  |  |  |  |
| Singh 2000 |  |  |  |  |  |  |  |  |  |  |  |  |  |  |  |  |  |  |  |  |  |  |  |  |
| Sirinavin 1998 |  |  |  |  |  |  |  |  |  |  |  |  |  |  |  |  |  |  |  |  |  |  |  |  |
| Skaer 1993 |  |  |  |  |  |  |  |  |  |  |  |  |  |  |  |  |  |  |  |  |  |  |  |  |
| Solomon 2001 |  |  |  |  |  |  |  |  |  |  |  |  |  |  |  |  |  |  |  |  |  |  |  |  |
| Sondergaard 2003 |  |  |  |  |  |  |  |  |  |  |  |  |  |  |  |  |  |  |  |  |  |  |  |  |
| Suwangool 1991 |  |  |  |  |  |  |  |  |  |  |  |  |  |  |  |  |  |  |  |  |  |  |  |  |
| Thomas 2006 |  |  |  |  |  |  |  |  |  |  |  |  |  |  |  |  |  |  |  |  |  |  |  |  |
| Toltiz 1998 |  |  |  |  |  |  |  |  |  |  |  |  |  |  |  |  |  |  |  |  |  |  |  |  |
| Van Kasteren 2005 |  |  |  |  |  |  |  |  |  |  |  |  |  |  |  |  |  |  |  |  |  |  |  |  |
| Verstappen 2004 |  |  |  |  |  |  |  |  |  |  |  |  |  |  |  |  |  |  |  |  |  |  |  |  |
| Verstappen 2004a |  |  |  |  |  |  |  |  |  |  |  |  |  |  |  |  |  |  |  |  |  |  |  |  |
| Verstappen 2003 |  |  |  |  |  |  |  |  |  |  |  |  |  |  |  |  |  |  |  |  |  |  |  |  |
| Wilson 1991 |  |  |  |  |  |  |  |  |  |  |  |  |  |  |  |  |  |  |  |  |  |  |  |  |
| Winkens 1995 |  |  |  |  |  |  |  |  |  |  |  |  |  |  |  |  |  |  |  |  |  |  |  |  |
| Winkens 1995a |  |  |  |  |  |  |  |  |  |  |  |  |  |  |  |  |  |  |  |  |  |  |  |  |
| Wones 1987 |  |  |  |  |  |  |  |  |  |  |  |  |  |  |  |  |  |  |  |  |  |  |  |  |
| Woodward 1987 |  |  |  |  |  |  |  |  |  |  |  |  |  |  |  |  |  |  |  |  |  |  |  |  |
| Young 1985 |  |  |  |  |  |  |  |  |  |  |  |  |  |  |  |  |  |  |  |  |  |  |  |  |
| Zwar 1999 |  |  |  |  |  |  |  |  |  |  |  |  |  |  |  |  |  |  |  |  |  |  |  |  |
